# The Deceptively Simple N170 Reflects Network Information Processing Mechanisms Involving Visual Feature Coding and Transfer Across Hemispheres

**DOI:** 10.1093/cercor/bhw196

**Published:** 2016-10-17

**Authors:** Robin A. A. Ince, Katarzyna Jaworska, Joachim Gross, Stefano Panzeri, Nicola J. van Rijsbergen, Guillaume A. Rousselet, Philippe G. Schyns

**Affiliations:** 1 Institute of Neuroscience and Psychology, University of Glasgow, Glasgow G12 8QB, UK; 2 Laboratory of Neural Computation, Istituto Italiano di Tecnologia, Rovereto 38068, Italy

**Keywords:** EEG, mutual information, information transmission, face processing, reverse correlation

## Abstract

A key to understanding visual cognition is to determine “where”, “when”, and “how” brain responses reflect the processing of the specific visual features that modulate categorization behavior—the “what”. The N170 is the earliest Event-Related Potential (ERP) that preferentially responds to faces. Here, we demonstrate that a paradigmatic shift is necessary to interpret the N170 as the product of an information processing network that dynamically codes and transfers face features across hemispheres, rather than as a local stimulus-driven event. Reverse-correlation methods coupled with information-theoretic analyses revealed that visibility of the eyes influences face detection behavior. The N170 initially reflects coding of the behaviorally relevant eye contralateral to the sensor, followed by a causal communication of the other eye from the other hemisphere. These findings demonstrate that the deceptively simple N170 ERP hides a complex network information processing mechanism involving initial coding and subsequent cross-hemispheric transfer of visual features.

## Introduction

The ultimate goal of cognitive neuroscience is to understand the brain as an organ of information processing. We subscribe to the assumption that the information processing systems of the brain, like all information processing systems, can be fruitfully described at different levels of abstraction, with specific contributions from different levels of granularity of brain signals (Marr 1982; Tanenbaum and Austin 2012). However, analysis at any level of abstraction will remain difficult unless we understand more directly what information the brain processes when it categorizes the external world. For example, our brain can quickly detect the presence of a face, implying that brain networks can extract and process the specific visual information required for face detection. As experimenters, we typically do not have a detailed description of such task-specific information and so we cannot explicitly test hypotheses about its algorithmic processing in brain signals.

Here, we address this issue by first isolating what specific information modulates face detection behavior. Then we examine, where, when, and how this face information modulates dynamic signals of integrated brain activity on the left and right hemispheres. Since neural activity produces these integrated signals, from them we can derive the timing and approximate regions where neural populations are processing the specific face information underlying behavioral responses.

**Figure 1.**
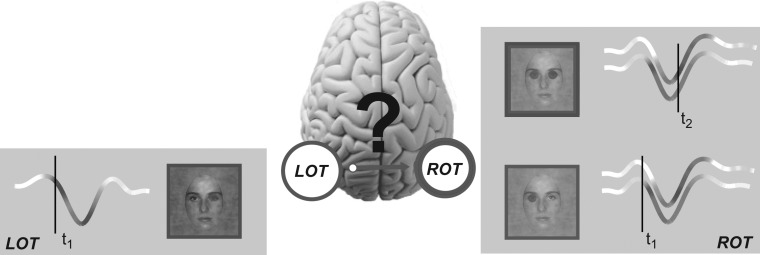
Hypothesis of cross-hemisphere feature transfer along the N170 time course*.* At time *t*_1_, the Left Occipito Temporal (LOT) and the Right Occipito Temporal (ROT) sensors reflect coding of the contralateral right and left eye, respectively. Coding strength is represented with variations of hue (blue for the left eye; red for the right eye) directly on the LOT and ROT N170 ERP waveforms. At a later time *t*_2_, ROT also codes the ispilateral right eye. The anatomy of the visual system suggests that this sensitivity could arise from cross-hemisphere transfer from LOT, where the right eye is coded at *t*_1_.

In humans, the N170 is the first integrated measure of cortical activity that preferentially responds to faces, with larger amplitudes to entire faces than to stimuli from other categories (Bentin et al. 1996; Rossion and Jacques 2008). We developed this account, demonstrating that the N170 waveform reflects a feature coding mechanism (Schyns et al. 2003; Smith et al. 2004; Schyns et al. 2007; van Rijsbergen and Schyns 2009; Rousselet et al. 2014a). With face stimuli, coding starts with the eye contralateral to the recording sensor (e.g., the left eye on the right sensor, see Fig. 1), on the downward slope of the N170 (~140 ms post-stimulus), followed in some face categorizations by the coding of task-relevant features—for example, coding of the diagnostic contralateral wrinkled nose corner in “disgust,” or the diagnostic corner of the wide-opened mouth in “happy”. By coding, we refer to the N170 time windows when the single-trial visibility of face features—randomly sampled with the Bubbles procedure, which randomly positions a number of Gaussian apertures on each trial to sample contiguous pixels from the face stimuli—covaries with the corresponding single-trial electroencephalography (EEG) responses.

Thus, converging evidence from face detection and categorization reveals that early face coding on the N170 differs between the left and right hemispheres. Specifically, as illustrated in Figure 1, at time *t*_1_ the right eye (represented in red) is initially coded on the left hemisphere N170, and the left eye (represented in blue) is coded on the right hemisphere N170 (Smith et al. 2007; Rousselet et al. 2014a). Furthermore, the later part of the N170 waveform additionally codes the eye ipsilateral to the sensor at time *t*_2_—i.e., the right eye on the right sensor; the left eye on the left sensor (Smith et al. 2007; Rousselet et al. 2014a).

We know from anatomy and physiology that the visual system is lateralized across two hemispheres, with a separate visual hierarchy in each that processes the contralateral visual hemifield, from early to higher order visual areas, where processing becomes bi-lateral (Essen et al. 1982; Clarke and Miklossy 1990; Saenz and Fine 2010). Could the later N170 ipsilateral eye coding at *t*_2_ reflect the transfer of specific features coded at *t*_1_, across the hemispheres, through to high-level visual areas?

Figure 1 illustrates our hypothesis in the context of a face detection task, where the eyes (predominantly the left one) modulate reaction times (Rousselet et al. 2014a). Panels LOT and ROT (for Left and Right Occipito-Temporal sensors, respectively) illustrate initial coding of the contralateral eye at early time *t*_1_, closely followed by coding of the eye ipsilateral to the sensor at a later time *t*_2_—for example, the right eye on ROT. Later coding of the right eye at *t*_2_ could arise from a causal transfer from its earlier coding at *t*_1_ on LOT on the opposite hemisphere. A demonstration of such feature transfer would suggest a reinterpretation of the N170, from a local event often interpreted as coding the entire face (Bentin et al. 1996; Eimer 2000), to the reflection of a more global information processing network that spans both hemispheres across multiple stages of face coding in the visual hierarchy. Here, we demonstrate that the N170 does indeed reflect network-level information processing mechanisms.

**Figure 2.**
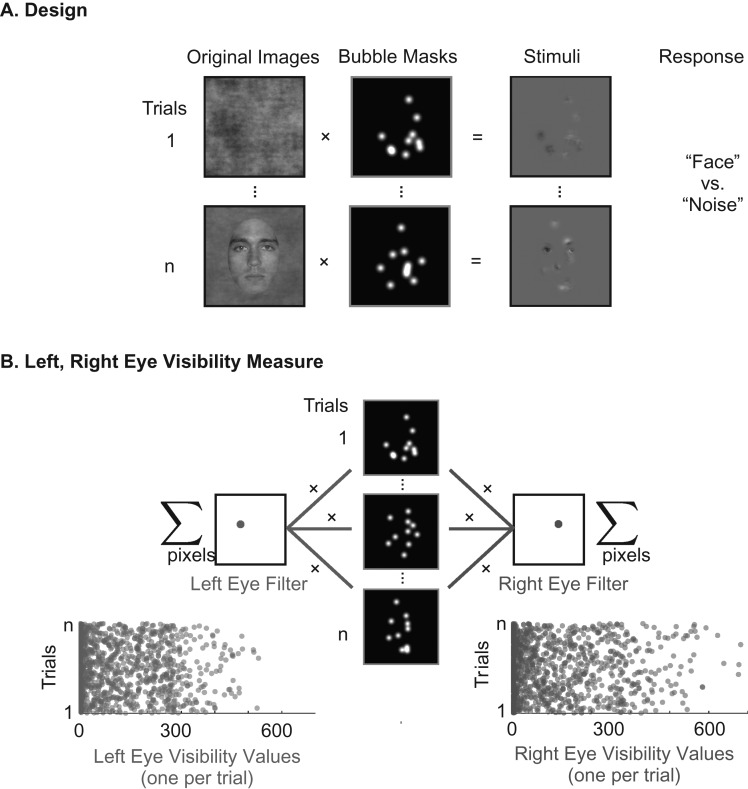
Bubble sampling and eye visibility. (*A*) Design: On each trial, we used a bubble mask comprising 10 Gaussian apertures to sample visual information from either a texture or a face image. Observers pressed a key to indicate which they detected (“face” or “noise”). (*B*) Left, Right Eye Visibility Measure: For each trial, we applied to the bubble mask a filter covering the spatial region of the left eye (left eye filter) and the right eye (right eye filter), counting the number of pixels the bubbles revealed within each regions. This produced 2 scalar values per trial representing the visibility of the left and right eye, respectively.

In a face detection task (Rousselet et al. 2014a, 2014b), we instructed observers (*N* = 16) to detect on each trial the presence of a face sparsely and randomly sampled with small Gaussian apertures, see Figure 2*A* and Rousselet et al., (2014a). Half of the trials sampled face images; the remaining half sampled amplitude spectrum matched noise, to dissociate spatial attention to feature location from feature coding per se. We recorded each observers’ EEG and face detection responses [correct vs. incorrect and Reaction Times, RTs (Rousselet et al. 2014a, 2014b)].

## Results

### Behavior

Observers were both fast and accurate, median of the median RT = 376 ms, [range = 287, 492]; mean accuracy = 91%, [range = 84, 97]. We used Mutual Information (MI) to compute the association between the sampled pixels and observer detection responses (face vs. noise). This revealed a significant relationship between pixel variations and behavior indicating that the pixels representing the left eye region in the image are relevant for behavior (Rousselet et al. 2014a Fig. 3). Computation of MI between sampled face pixels and the more sensitive RT measure revealed that most observers responded faster on trials that revealed the left eye—a minority also responded faster to trials revealing the right eye (Rousselet et al. 2014a Fig. 3). On noise trials, MI values were low and not clustered on specific face features. Henceforth, we focus on the EEG coding and transfer of the eyes on face trials due to their prominence across observers in the face detection task (Rousselet et al. 2014a).

**Figure 3.**
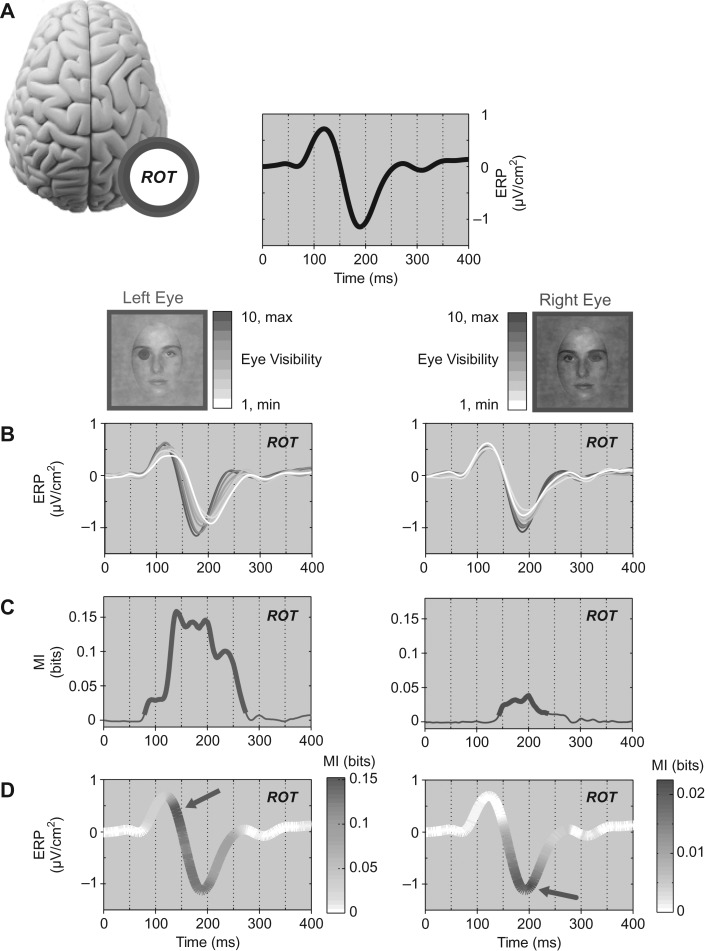
Coding. (*A*) The ERP measured on ROT for a typical observer (black curve). For this observer, ROT was selected as electrode B9 in the Biosemi coordinate system, which is posterior to PO8 (on the same radial axis). The left eye and right eye schematics illustrate with their color-coded blue and red scales the deciles of eye visibility across experimental trials (with 10 = highest eye visibility trials and 1 = lowest). (*B*) We recomputed ROT ERPs using only the trials from each decile of left and right eye visibility, to illustrate how eye visibility modulates ERPs. (*C*) CMI between left eye (blue curve) and right eye (red curve) visibility and corresponding ROT EEG response (a thicker line indicates regions of statistically significant MI, referred to as left and right eye coding). (*D*) CMI curves visualized over the ERP curve to precisely indicate when, over the time course of the ERP, eye coding peaks (indicated with a color-coded arrow). Across observers and stimulus features we found 26 instances (out of 30 = 15 observers × left/right eye) of significant eye CMI on both contralateral and ipsilateral sensors.

### Electroencephalography

We removed one observer from analysis due to poor EEG signal (see Materials and Methods). For the remaining 15 observers and for each sensor we computed the sensitivity of the EEG to the left and right eyes as follows. First, we used a mask for the left and the right eye regions and summed the Gaussian apertures within each eye region. The resulting two scalar values represent the visibility of each eye on each trial (Fig. 2*B*). Then, across trials we quantified the relationship between eye visibility values and the Current Source Density (CSD) EEG measured at each sensor and time point. To remove from the quantification any effects of weak statistical dependence between the left and right eye sampling, we calculated Conditional Mutual Information (CMI) between each eye and the EEG response, conditioning out any effect of the visibility of the alternate eye. Henceforth, we refer to these effect sizes interchangeably as CMI time courses or eye coding curves. On each hemisphere, we then identified the single occipital-temporal sensor (i.e., LOT and ROT) with largest CMI to the contralateral eye within the N170 time window (100–200 ms)—see Materials and Methods.

To address our hypothesis (cf. Fig. 1), we propose three requirements that are necessary for the existence of a causal transfer of stimulus features between two brain regions. The first requirement is “coding”: Both regions should code the same stimulus feature (e.g., the right eye at *t*_1_ on the LOT N170; the right eye at *t*_2_ on the ROT N170). The second requirement is “temporal precedence”: Feature coding in the first region should occur before coding of the same feature in the other region (e.g., the right eye on LOT N170 at *t*_1_ and the right eye on ROT N170 at *t*_2_). Finally, there should be what we term “coding equivalence”. The information about the right eye that an external observer could extract from LOT at *t*_1_ on individual trials should be the same (or at least highly similar) to that an observer could extract from ROT at *t*_2_. This requirement is crucial to infer that coding of the right eye at *t*_1_ on LOT is transmitted to ROT at *t*_2_. Without it, we could not rule out that coding of the right eye on ROT is independent from that at LOT, and hence not the result of communication.

### Coding

To operationalize coding, we refer to the CMI time courses of the chosen LOT and ROT sensors for the left and right eye. Figure 3 illustrates this analysis for one observer. For reference, the black curve shows a typical N170 obtained on the ROT sensor (Fig. 3*A*). Standard interpretations would consider this average as a local response to full-face stimuli, in contrast to other categories (Bentin et al. 1996; Eimer 2000). Here, random sampling with bubbles changes the visibility of the left and right eyes across trials (Fig. 2*B*) and so we can analyze how eye visibility modulates single-trial N170 responses. On the ROT N170, we split the trials into 10 bins of left eye visibility (deciles of the distribution across trials; represented with shades of blue, Fig. 3*A*) and right eye visibility (represented with shades of red). For each bin we computed and plotted the mean ERP. Figure 3*B* illustrates that increased left eye visibility causes an earlier and larger N170. Increased visibility of the right eye caused larger N170 amplitude, with no change in latency.

Plotting the ERP for each decile of eye visibility demonstrates a modulation. To quantify this coding, we calculated, for each eye, the CMI between eye visibility and the corresponding EEG response at each time point at LOT and ROT sensors (see Methods). In Figure 3*C*, CMI time courses indicate with a thicker line the time windows of a statistically significant relationship (*p*=0.01, corrected for multiple comparison over time [0–400 ms], all sensors and the two eye features with the method of maximum statistics). CMI time courses show that in this observer the ROT N170 codes the left and right eyes. All 15 observers showed significant CMI on the contralateral sensor for at least one eye (13/15 significant for both eyes). We found 14/15 observers also showed significant CMI of the eye ipsilateral to the sensor, for at least one eye (13/15 significant for both eyes). These results satisfy the coding requirement because across observers and stimulus features we found 26 instances (out of 30 = 15 subjects × left/right eye) of significant eye CMI on both contralateral and ipsilateral sensors.

### Temporal Precedence

Figure 3*D* reports the eye coding CMI curves superimposed as hue on the ROT ERP time course, to directly visualize the timing of left and right eye coding on this sensor. The ROT N170 codes the contralateral left eye throughout, with a strongest early effect at the onset of the negative deflection (see blue arrow). The ROT N170 also codes the ipsilateral right eye, but later, with the strongest effect just after the N170 peak (see red arrow). This illustrates that the ROT N170 codes the contralateral left eye before the ipsilateral right eye.

**Figure 4.**
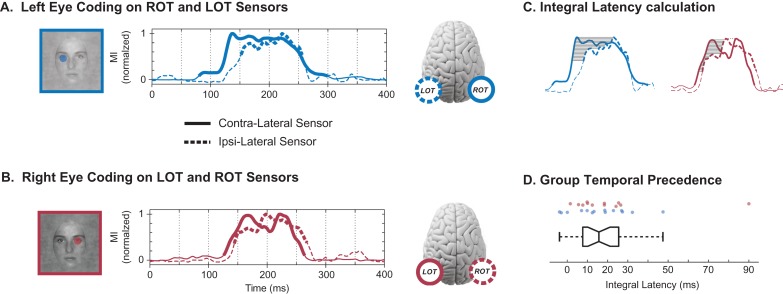
Temporal precedence*. *(*A*) Left Eye Coding on ROT and LOT Sensors: The peak-normalized CMI curves indicate the initial coding of the left eye on the contralateral ROT sensor (plain curve) and the later coding of the same eye on the ipsilateral LOT sensor (dashed curve). Thicker sections of the MI curves represent statistical significance. (*B*) Right Eye Coding on LOT and ROT Sensors: The normalized MI curves indicate the initial coding of the right eye on the LOT sensor (plain curve) and the later coding of the same eye on the ROT sensor (dashed curve). Thicker sections of the MI curves represent statistical significance. (*C*) Integral Latency Calculation: We quantified temporal precedence with an integral latency measure. We normalized the CMI curves to their peak values (left eye, blue; right eye, red) and calculated the average delay between the CMI curves of the contralateral and ipsilateral sensors (solid, dashed lines respectively), over the *y*-axis region where both were significant (shaded region). (*D*) Group Temporal Precedence: Box plot of the integral latency (in ms) between contralateral and ispilateral coding of the same eye across hemispheric sensors. Positive values correspond to earlier contralateral coding. Each dot above represents a particular observer and eye feature—blue for left eye (ROT to LOT latency), red for right eye (LOT to ROT latency).

We now demonstrate the temporal precedence of the left (or right) eye across the contra and ipsilateral sensors. To visualize this comparison, Figure 4*A* reveals the peak-normalized CMI time courses of the left eye on ROT (plain blue line) and LOT (dashed blue line); Figure 4*B* presents the CMI of the right eye on LOT (plain red line) and ROT (dashed red line). Comparison of the solid (contralateral sensor) to the dashed (ipsilateral sensor) CMI curves illustrates contral-lateral temporal precedence in both cases. To quantify temporal precedence, we considered each instance (specific observer and eye feature) with significant same eye coding across the contralateral and ipsilateral sensors (*N* = 26). For each instance, we quantified the coding latency by normalizing the CMI curves to their peak values and calculating the average delay between the CMI curves over the *y*-axis region where both were significant (gray region, Fig. 4*C*; see Materials and Methods). We refer to this measure as “integral latency”. At the group level, observers showed a significant integral latency of same eye coding on the contralateral before the ipsilateral sensor (Fig. 4*D*; Sign Rank test, *P* = 7.4e^−7^). The median contra-to-ipsi latency was 15.7 ms (first quartile = 7.7, third quartile = 25.5 ms). 13/15 observers had significant bilateral coding with a contra-ipsi latency of greater than 7 ms for at least one eye feature. There was no difference in contra-to-ipsi latencies between left eye (ROT to LOT; median 15.8 ms, IQR [7.1 26.9] ms) and right eye (LOT to ROT; median 15.3 ms, IQR [8.7 24.8] ms). Across observers, we have now established temporal precedence of same eye coding from the contralateral to the ipsilateral sensor.

### Coding Equivalence

**Figure 5.**
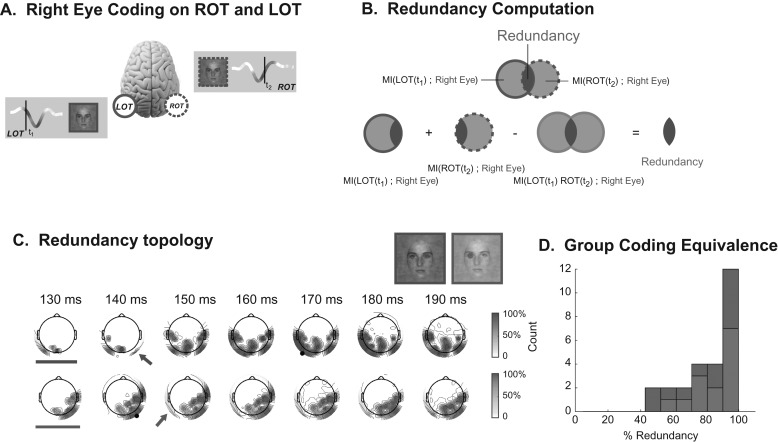
Coding equivalence. (*A*) Illustration of the coding redundancy of the right eye between early LOT and late ROT: Coding redundancy of the right eye is computed between LOT and ROT at the peak of each MI curve (i.e., at *t*_1_ on LOT and *t*_2_ on ROT). (*B*) Redundancy Computation: Venn diagrams illustrate the computation of redundant (i.e., intersecting) eye information on LOT and ROT. (*C*) Redundancy Topography: Using as seed the peak MI time point on LOT (marked with black circle on red topographies), and ROT (black circle on blue topographies) the time courses of redundancy topographies illustrate when and where (see color-coded arrow) redundant ipsilateral coding of the eyes begins. Note that for both eyes, the 130 ms time point (indicated with bar) shows no ipsilateral redundancy. (*D*) Group Coding Equivalence: Left (in blue) and right (in red) eye coding redundancy between LOT and ROT, at the respective peaks of the MI curves, expressed as percentages.

We turn to coding equivalence, the third and final necessary condition for causal feature transfer. Figure 5*A* schematizes our results so far. We know that the LOT N170 codes the contralateral right eye at *t*_1_—this is represented with plain lines. We also know that the ROT N170 codes the same eye at a later time, *t*_2_—this is represented with dashed lines. Now, we establish that the eye feature coded at these two different time points and on different sensors is mostly equivalent.

To provide an intuitive understanding of coding equivalence, consider Figure 5*A* and a putative observer who would read out only the early EEG responses from the LOT sensor. Could they predict the visibility of the right eye on each trial? Would adding information from the later EEG responses on ROT improve their prediction? If the early LOT and later ROT information were the same, adding the later ROT responses would not add any new knowledge about right eye visibility and thus would not improve prediction. However, if adding ROT information did improve prediction, then ROT responses would contain extra information about the visibility of the eye that is not already available in LOT responses, indicating that ROT and LOT coding of the right eye were not equivalent.

We formalized such coding equivalence with an information theoretic quantity called “redundancy” illustrated in Figure 5*B* (see Methods). Venn diagrams represent the CMI (at peak time) to the right eye on LOT (plain line) and ROT (dashed line). Coding equivalence corresponds to their overlap (i.e., their intersection). Because CMI is additive we measure this overlap directly, with a simple linear combination of CMI quantities. We sum the LOT and ROT CMI separately (which counts the overlapping CMI twice) and subtract the CMI obtained when LOT and ROT responses are considered together (which counts the overlap once). The resulting redundancy value measures coding equivalence—that is, the CMI that is shared between the LOT and ROT responses.

For each observer, we computed redundancy between the N170 CMI peaks for the left eye (a transfer from right to left hemispheres) and for the right eye (the opposite transfer, from left to right hemispheres). Figure 5*C* illustrates the redundancy topography time courses for one typical observer. Redundancy is computed with respect to a seed response—a particular time point (CMI peak) of either LOT (for right eye redundancy, red topographies, seed indicated with black marker) or ROT (for left eye redundancy, blue topographies, seed indicated with black marker). At 130 ms there is contralateral, but no ipsilateral coding of either eye (indicated with bars); ipsilateral redundant coding appears from 10 to 20 ms later (arrows). The redundancy topographies also illustrate that our analyses do not depend on the choice of a single seed sensor in each hemisphere. Figure 5*D* shows a histogram of the normalized redundancy over all 15 observers. In almost all cases redundancy is large (median = 88%) demonstrating the high coding similarity of the same eye across hemispheric locations and time segments of the N170. 14/15 observers have contra-ipsi redundancy greater than 50% for at least one eye feature (21/26 bi-lateral instances).

To provide further evidence that delayed redundancy across hemispheres actually represents feature transfer we now demonstrate that the two hemispheres exchange information with each other, above and beyond that related to stimulus features. To this end, we calculated the CMI between LOT and ROT peaks directly, removing (i.e., conditioning out) the effect of variability of both eye features. These values were large (compared with stimulus feature coding) and significant. All 26 instances showed significant CMI: median = 0.32 bits, min = 0.07 bits, max = 0.65 bits; median of the lower boundaries of the 99% bootstrap confidence interval = 0.25 bits, min = 0.04 bits, max = 0.55 bits). This demonstrates that there is a time-delayed relationship between LOT and ROT on single trials, unrelated to eye visibility, over and above the relationship that could be expected if these two regions received a common eye feature signal from a third region but did not exchange information with each other. This can be interpreted as causal transfer within the Wiener–Granger framework (Wiener 1956; Granger 1969; Bressler and Seth 2011), and so shows that our observations are better explained by a model that includes direct communication between the regions, rather than a model involving a third region that sends the same eye signal with a different delay to LOT and ROT, but in which LOT and ROT are not directly connected.

## Discussion

In a face detection task, we derived an abstract, high-level information processing interpretation of brain activity. First, using the Bubbles procedure coupled with behavioral responses, we showed what information supports face detection: the eyes of a face. Then, with the Bubbles procedure coupled with CSD transformed EEG data to improve spatial localization (Tenke and Kayser 2012), we reconstructed a network revealing that the sources generating the N170 code and transfer the eyes of a face. We specified three necessary conditions for inferring feature transfer within our measurement paradigm: coding of the same features in two brain regions, temporal precedence of coding in one region with respect to the other and coding equivalence of the feature representation in the two regions. Our analyses revealed that 13/15 observers individually meet all three conditions. Specifically, in each observer we demonstrated coding of the same eye on the LOT and ROT sensors. We showed the temporal precedence of same eye coding at contralateral before ipsilateral sensors. Finally, we showed high redundancy (i.e., high coding similarity) between early and late coding of the same eye on opposite hemispheres. Together, these three conditions suggest causal transmission of eye information in the Wiener–Granger sense (e.g., right eye from the early part of the LOT N170 to late part of the ROT N170). Though we selected one LOT and one ROT electrode per observer for statistical analyses, the topographic maps of Figures S2–S16 reveal that the three conditions would be met by choosing other electrodes from the extended lateral regions of delayed redundancy across hemispheres. The clear separation between the clusters of redundant information on the left and right hemispheres further supports the claim that LOT and ROT signals originate from different brain regions.

The N170 now appears as a deceptively simple signal that actually reflects an inter-hemispheric information processing network that codes and communicates face features. A distinctive feature of our approach is that we are not just reconstructing a network on the basis that two brain regions communicate; we are revealing “what they are communicating about”—that is, the information content underlying face detection. It is the novel quantification of the content of information coding and transfer that represents an important step towards a new brain algorithmics to model the information processing mechanisms of perception and cognition (Schyns et al. 2009). This is a radical departure from typical N170 quantifications and interpretations, which compare peak amplitude and latency differences between the average EEG responses to a few categories comprising complete stimulus images (Bentin et al. 1996; Rossion and Jacques 2008). In contrast, here we decompose the stimulus with Bubbles to test the N170 responses against random samples of stimulus pixels presented on each trial. This enables a precise characterization of the information subtending the task and the dynamics of coding alongside the N170 time course in individual observers. We demonstrated that focusing on the average N170 peak was restricting interpretation of the N170 signals because 1) feature coding is represented in the single trial variance of the N170 (not its mean), revealing that 2) contralateral eye coding starts on the N170 downward slope, ~40 ms prior its peak (see also Schyns et al. 2007) and that 3) the rebound from the peak codes the transferred ipsilateral eye from the other hemisphere. Together, our results demonstrate that the N170 cannot be fully interpreted as an isolated event on one hemisphere. Rather, it reflects the coding and communication in an information processing network involving both hemispheres.

### Feature Transmission

Our results provide strong evidence for causal feature communication between hemispheres. We review the evidence in turn. First, the lateralized anatomy of the visual system prescribes that the first cortical processing of each eye should occur in the early part of the contralateral visual hierarchy. This is congruent with our findings. As there is no direct anatomical pathway from the ipsilateral hemifield (only a contralateral pathway), the ipsilateral eye information present in the second part of the LOT and ROT N170 should be transferred from the opposite hemisphere. We demonstrated this functionally, without recourse to anatomical priors in our analysis. Second, the latency timings of inter-hemispheric feature transfer (median 15.7 ms) are consistent with previously reported inter-hemispheric transfer times (Brown et al. 1994; Ipata et al. 1997). Third, we demonstrate that the coding redundancy between LOT and ROT occurs in the context of single-trial temporal relationships between these regions that cannot be explained simply by a third region sending a common eye feature signal with a different delay. In sum, the reported evidence is consistent with communication between the two hemispheres, a proportion of which is “about” the eye.

A previous analysis of this dataset has shown that the effects of the two eyes are not equal; there is a lateral asymmetry with the left eye having systematically stronger effects both on behavior, and on the contralateral EEG signal, compared with the right eye (Fig. 3*C* and Rousselet et al. 2014a). Although we do not find any systematic asymmetry in terms of latency or normalized redundancy, the asymmetry in MI means that in absolute terms the communication we infer about the left eye (from ROT to LOT) will be greater in amplitude than that about the right eye (from LOT to ROT). As we are able to measure statistically significant causal communication even for the weaker representation of the right eye, we do not see any qualitative asymmetry in the information processing network, however the quantitative differences should be kept in mind.

Note that our approach represents a major conceptual shift compared with traditional measures of functional connectivity, such as Granger causality (Granger 1969; Bressler and Seth 2011), transfer entropy (or directed information) (Massey 1990; Schreiber 2000; Wibral et al. 2014), and other measures (Bastos and Schoffelen 2016). These approaches quantify causal statistical relationships between the activity of different brain regions, without considering whether there is communication about specific external stimulus features. We recently developed Directed Feature Information (DFI) (Ince et al. 2015), a novel measure of functional connectivity that quantifies communication about a specific stimulus feature between different brain regions. This time domain measure calculates such communication with a delay that is explored parametrically, while conditioning out the activity of the receiving area at the same delay. Here, this time domain approach was difficult to apply because the inter-hemispheric delays we report are short compared with the autocorrelation of the low-pass filtered EEG signal. With the DFI measure, when conditioning out the auto-correlated past of the receiving sensor at such short delays, we did not have sufficient signal to noise to detect the stimulus feature communication. For this study, we therefore developed the peak-to-peak analysis presented here, which contains the core logical elements of the Wiener–Granger framework extended with a measure of similarity of information content, which, analogous to DFI, allows us to evaluate if the observed functional connectivity includes communication about a stimulus feature.

### Sources Generating the N170

Further research will seek to reduce the abstract information dynamics presented here. We will examine the networks of brain sources that generate the left and right hemisphere N170s and implement the information processing functions of task-dependent contralateral and ipsilateral feature coding and cross-hemispheric transfer. In our data, topographic maps of contralateral eye coding suggest the involvement of posterior-lateral sources. Source analyses or correlations between BOLD and ERP amplitudes suggest that the N170 sources locate around the STS (Watanabe et al. 2003; Itier and Taylor 2004; Sato et al. 2008; Nguyen and Cunnington 2014), the fusiform gyrus (Horovitz et al. 2004), or both (Sadeh et al. 2010; Dalrymple et al. 2011; Prieto et al. 2011). To better localize these sources and understand how they code and transfer task-relevant features, we could apply our bubbles paradigm with single-trial fMRI-EEG measures. In fact, an MEG reverse-correlation study revealed coding of face features, including the eyes, in the time window of the M170 in lateral cortical areas (Smith et al. 2009). Finally, intracranial data also support the involvement of occipital and temporal lateral areas, such as the right inferior occipital gyrus, to generate scalp N1/N170 (Sehatpour et al. 2008; Rosburg et al. 2010; Engell and McCarthy 2011; Jonas et al. 2012, 2014). So, lateral sources are likely to be involved in the generation of the N170. The information processing mechanisms revealed here guide their study at the source level, in terms of the timing of coding and transfer of specific features.

### Advantages of Information Theory for Analysis of Brain Signals

It is often thought that the EEG signal is too noisy for single-trial analyses as done here. It is worth noting that several novel methodological developments delivered important advances. First, a copula-based MI estimator provided a flexible and powerful multivariate statistical framework to quantify feature coding with the full temporal resolution of EEG (Ince et al. 2016). Second, as an improved measure of EEG activity, we considered a bivariate response consisting of the recorded voltage at each time point together with the temporal gradient (see Methods and Figure S1). Third, to eliminate effects of the alternative eye when computing eye coding, we used conditional MI throughout our analyses. CMI is a powerful and rigorous approach to quantify feature coding when stimulus features are correlated, and is applicable in any sensory modality. Finally, the additivity of CMI enables a direct quantification of feature coding interactions between different regions of the brain and time points of their activity. Here, we used CMI additivity to compute redundancy, but the same computation can also reveal synergistic interactions. Using CMI to quantify coding redundancy and synergy has broad applications in neuroimaging, from quantifying the interactions between different spatial regions and time points to construct brain representations (as considered here), to quantifying the relationships between signals from different brain imaging modalities (e.g., EEG/fMRI).

### Hierarchical Analysis of Information Processing

Our interpretative approach can be applied hierarchically, to different levels of response granularity (e.g., from behavior to EEG to neurons), to quantify information processing mechanisms at each level. At the coarsest level, with behavioral measures (accuracy and RT) we determine what visual features the organism processes to discriminate faces from noise—that is, the two eyes. This reduces the full high-dimensional stimulus to a few, lower-dimensional task-relevant features. Going down the hierarchy, with integrated EEG measures we determine where, when, and how states of brain activity (e.g., variance of the early and late parts of the single-trial N170 signals) code and transfer task-relevant features. That is, we relate information processing to a few states of brain activity (e.g., coding of the contralateral eye during the early part of the ROT and LOT N170; coding of the ipsilateral eye over the later part). We also relate operations on information (e.g., transfer of the ipsilateral eye across hemispheres) to brain state transitions (e.g., eye transfer occurs between the early and late part of the LOT and ROT N170s). Experimentally, we can repeat the exercise one (or several) hierarchical level(s) down, for instance measuring the localized MEG sources that generate the N170, to add the details of information processing that would be reverse engineered from the finer grained measures of neural activity. We expect these more detailed information processing descriptions to be consistent with the results described here.

The critical point is that while the information processing ontology produces finer information processing details with increasing granularity of brain measures, it preserves the information gained from analyses at more abstract levels (as shown here between behavior and the EEG that preserves the eyes). Abstract levels guide the search for detailed implementation of the behaviorally relevant information (e.g., contra- and ipsi-later eyes) and functions (e.g., coding, transfer) in the increased complexity of the lower levels—that is, whatever else the neural populations in the sources of the N170 may be encoding, the population must be sensitive to first the contralateral and then the ipsilateral eye and transfer of the latter should come from a population on the other hemisphere. Our approach is similar to analyzing a computing architecture hierarchically across levels, from the most abstract (e.g., “send mail”), to its programming language algorithm, to its assembly language, to its machine-specific implementation. The critical difference between a brain and a computer is that whereas we can directly engineer (via a hierarchy of compilers) an abstract information algorithm into a computer hardware, we can only reverse engineer an information processing algorithm from brain data to infer a hierarchy.

### Influences of Categorization Tasks

Our approach will be particularly interesting when applied to study the processing of the same stimuli when the observer is performing different tasks. For example, with multiple categorizations of the same faces (e.g., gender, expression, and identity), we could determine from behavior the specific features that are relevant for each task (the what) and then trace, as we have done here, where and when each feature set is coded and transferred between localized brain sources (e.g., with MEG Smith et al. 2009). As the task and the associated behaviorally relevant information changes, we can determine how the corresponding processing in brain networks is affected: Where and when are task-relevant features coded and task-irrelevant features suppressed? This is a pre-requisite to addressing elusive questions such as the locus of selective attention, the role of top-down priors, and their influence on the construction of the information content of stimulus perception and categorization. How does network feature communication change with task? Again, this is an important condition to understand, for example, information integration. Our results propose a specific timing of feature coding and transfer that could constrain the study of feature integration mechanisms—specifically, these should occur after transfer of the contra-laterally coded features, possibly in occipito-temporal sources, before decision-making processes (Smith et al. 2004; Philiastides and Sajda 2006).

Here, by addressing the what, where, when, and how questions of information processing, we proposed a radically new interpretation of the N170, as reflecting a network that codes and transfers a specific information content across hemispheres. The main methodological advantage of focusing on the what and then reducing its processing across the levels of an information processing ontology is akin to the main recommendation of Marr's computational analysis: The abstract information goals of the system guide the analysis. Revealing the information, from behavior to the processing states of the brain and their transitions brings us one step closer to the ultimate goal of cognitive neuroimaging of understanding the brain as a machine that processes information.

## Materials and Methods

The data considered here were already reported in Rousselet et al., (2014a). Full experimental details are provided there. Data are available at Rousselet et al., (2014b).

### Observers

The study comprised 16 observers: 9 females, 15 right-handed, median age 23 (min 20, max 36). Prior to the experiment, all observers read a study information sheet and signed an informed consent form. The experiment was approved by the Glasgow University College of Science and Engineering Ethics Committee with approval no. CSE00740. All observers had normal or corrected-to-normal vision and contrast sensitivity of 1.95 and above (normal score).

### Stimuli

Stimuli were gray-scale pictures of faces and textures (Rousselet et al. 2014a Fig. 1). Faces from 10 identities were used; a unique image was presented on each trial by introducing noise (70% phase coherence) into the face images (Rousselet et al. 2008). Textures were face images with random phase (0% phase coherence). All stimuli had an amplitude spectrum set to the mean amplitude of all faces. All stimuli also had the same mean pixel intensity, 0.2 contrast variance and spanned 9.38 × 9.38 degrees of visual angle. The face oval was 4.98 × 7.08 degrees of visual angle. Face and noise pictures were revealed through 10 two-dimensional Gaussian apertures (sigma = 0.368 degrees) randomly positioned with the constraint that the center of each aperture remained in the face oval and was at a unique position. In the rest of this article, we refer to these masks with Gaussian apertures as bubble masks.

### Experimental Procedure

At the beginning of each of two experimental sessions, we fitted observers with a Biosemi head cap comprising 128 EEG electrodes. We instructed observers as to the task, including a request to minimize blinking and movements. We asked observers to detect images of faces and textures as fast and as accurately as possible. They pressed the numerical pad of a keyboard for response (“1” for face vs. “2” for texture) using the index and middle fingers of their dominant hand. Each experimental session comprised 1200 trials, presented in blocks of 100, including 100 practice trials. All observers participated in two experimental sessions lasting in total about 4 h and bringing the total number of trials per observer to 2200.

Each trial began with the presentation of a small black fixation cross (0.48 × 0.48 degrees of visual angle) displayed at the center of the monitor screen for a random time interval between 500 and 1000 ms, followed by a face or texture image presented for ~82 ms (7 refresh frames). A blank gray screen followed stimulus presentation until observer response.

### EEG Preprocessing

We removed one observer from analysis due to poor EEG signal. This was determined initially by visual inspection, but in addition, MI at all electrodes was flat, without departure from baseline.

EEG data were re-referenced offline to an average reference, band-pass filtered between 1 Hz and 30 Hz using a fourth order Butterworth filter, down-sampled to 500 Hz sampling rate and baseline corrected using the average activity between 300 ms pre-stimulus and stimulus presentation. Noisy electrodes and trials were detected by visual inspection on an observer-by-observer basis. We performed ICA to reduce blink and eye-movement artifacts, as implemented in the infomax algorithm from EEGLAB. Components representing blinks and eye movements were identified by visual inspection of their topographies, time courses, and amplitude spectra. After rejection of artefactual components (median = 4; min = 1; max = 10), we again performed baseline correction. Finally, we computed single-trial spherical spline CSD waveforms using the CSD toolbox with parameters iterations = 50, *m* = 4, lambda = 1.0e−5 (Kayser and Tenke 2006; Tenke and Kayser 2012). The CSD transformation is a second spatial derivative (Laplacian) of the EEG voltage over the scalp that sharpens ERP topographies and reduces the influence of volume-conducted activity. The head radius was set to 10 cm, so that the ERP units in all figures are μV/cm^2^. We also calculated the central-difference numerical temporal derivative of the CSD signal for each sensor and on each trial.

### Behavior Information: MI between Pixels and Detection and Reaction Time

Our analysis focuses on the single trial bubble masks because they control the visibility of the underlying image. Bubble masks take values between 0 and 1, controlling the relative opacity of the mask at each pixel, with 0 being completely opaque (pixel was shown gray) and 1 being completely translucent (pixel of underlying image was shown unaltered). We analyzed the bubble masks at a resolution of 192 × 134 pixels. For each observer and for each image pixel, we applied MI (Cover and Thomas 1991) to compute the relationship between single-trial pixel visibility and detection responses, and separately the relationship between single-trial pixel visibility and reaction times. We computed one MI pixel image for the face trials and a separate MI image for the texture trials. We found (Rousselet et al. 2014a Fig. 3) that the main face regions that systematically produced an effect on behavior were the eyes of the face images, but there was no such effect on texture trials. In the ensuing EEG analyses, we therefore only considered the face trials.

### EEG Information: MI between Eye Visibility and EEG Sensor Response

As the eyes are the critical regions affecting behavioral measures, we reduced the dimensionality of the single trial bubble masks to a measure of the visibility of each eye, to directly track their coding in the EEG. To this aim, we manually specified 2 circular spatial filters, one to cover each eye region (determined from the mean face image). We applied each of these filters to the bubble mask on each trial to derive a scalar value representing the visibility of the left eye and another scalar value representing the visibility of the right eye. Specifically, for each trial we sum the total bubble mask visibility within the left eye region (and separately for the right eye region). These 2 scalars represent the area of each eye that is visible on that trial (Fig. 2*B*). We used the same eye region filters for all observers.

For each observer, we then used CMI to quantify the relationship between the scalar visibility of each eye on each trial and the corresponding EEG signal, on each electrode (median 118, range 98–125). Within the framework of information theory, MI is a statistical quantity that measures the strength of the dependence (linear or not) between 2 random variables. It can also be viewed as the effect size for a statistical test of independence. One advantage of MI is that it can be applied with multivariate response variables, as done here. For each sensor and time point we considered a bivariate EEG response comprising the raw voltage value and the instantaneous gradient (the temporal derivative of the raw EEG). Supplementary Figure S1 illustrates with an example the smoothing effect of including the instantaneous gradient in the MI calculation. The top plot reproduces the panel of Figure 3 showing how visibility of the left eye modulates the ERP waveform. The rank correlation plot illustrates rank correlations between left eye visibility and EEG voltage measured at each time point. Correlation is a signed quantity that reveals transitions between regions of positive correlations (when ERP voltage increases with increased eye visibility) and regions of negative correlations (when ERP voltage decreases with increased eye visibility). At each transition (zero crossing), there is no measurable effect in the raw EEG voltage, even though this time point is within the time window when eye visibility clearly affects the ERP waveform. With MI, we addressed this shortcoming by computing at each time point the relationship between eye visibility and the bivariate EEG response comprising the raw EEG voltage and its instantaneous gradient. The 2 bottom MI plots demonstrate that the effect of adding the instantaneous gradient to smooth out the transitions and provide a more faithful and interpretable measure of coding dynamics.

The sampling strategy using bubbles (with fixed number of 10 bubble apertures per bubble mask on each trial) induces a weak dependence between the 2 (left and right) eye feature values across trials (median MI = 0.011 bits, range 0.0015–0.023 bits, significant at *P* = 0.01 uncorrected for 12/15 observers). This arises because a high visibility value of the left eye on a given trial indicates a high concentration of individual bubbles in that area, implying that fewer bubbles are distributed over the remainder of the face, including the right eye. This dependence introduces an ambiguity for interpretation. For example, if the EEG signal had a high left eye MI value and a low right eye MI value at a given time point, 2 interpretations would be possible. First, the EEG genuinely codes the right eye, irrespective of the visibility of the left eye. Second, the EEG only codes the left eye, and the low right eye MI value arises from the statistical dependence between the 2 eyes, as just discussed. We addressed this potential ambiguity with Conditional Mutual Information (CMI, the information theoretic analog of partial correlation) (Cover and Thomas 1991; Ince et al. 2012). CMI quantifies the relationship between any 2 variables (e.g., left eye visibility and the EEG response) while removing the common effect of a third variable (e.g., right eye visibility). We thus calculate CMI between left eye and the EEG signal, conditioned on the right eye (i.e., removing its effect), and similarly the CMI between the right eye and the EEG signal conditioned on the left eye (removing its effect).

We calculated CMI using a bin-less rank based approach based on copulas (Ince et al. 2015; Kayser et al. 2015; Ince et al. 2016). Due to its robustness this approach is particularly well suited for noisy continuous valued neuroimaging data such as EEG and provides greater statistical power than estimates based on binning. The following paragraphs detail this MI estimator. They can be skipped without loss of continuity.

A copula (Nelsen 2006) is a statistical structure that expresses the relationship between 2 random variables (e.g., between the left eye visibility and the EEG response on one electrode, at one time point). The negative entropy of a copula between 2 variables is equal to their MI (Ma and Sun 2011). On this basis, we fit a Gaussian copula to the empirical copula obtained from the eye visibility and EEG response, estimate its entropy and then obtain MI as the negative of this entropy. While this use of a Gaussian copula does impose a parametric assumption on the form of the interaction between the 2 variables, it does not impose any assumptions on the marginal distributions. This is important because the distribution of the visibility of the eye across trials is highly non-Gaussian. Since the Gaussian distribution is the maximum entropy distribution for a given mean and covariance, the Gaussian copula has higher entropy than any other parametric copula model that preserves those statistics. This MI estimation is therefore a lower bound on the true MI.

In practice, we calculated the empirical CDF values for a particular sensor and time point by ranking the data recorded across trials, and then scaling the ranks between 0 and 1. We then obtained the corresponding standardized value from the inverse CDF of a standard normal distribution. We performed this normalization separately for the EEG voltage and gradient, before concatenating them to form a 2D EEG response variable *R*. We computed CMI between these standardized variables using the analytic expressions for the entropy of univariate, bivariate, tri-, and quad-variate Gaussian variables (Misra et al. 2005; Magri et al. 2009):

$$I\left(S_{L};R|S_{R}\right)=H\left(S_{L},S_{R}\right)+H\left(S_{R},R\right)-H\left(S_{L},S_{R},R\right)-H\left(S_{R}\right)$$

We estimated entropy terms and corrected for the bias due to limited sampling using the analytic expressions for Gaussian variables (Misra et al. 2005; Magri et al. 2009). A particular advantage of this estimation method is its multivariate performance, which we exploit here with our 2D EEG voltage and gradient responses.

We determined statistical significance with a permutation approach, and addressed the problem of multiple comparisons using the method of maximum statistics (Holmes et al. 1996). For each of 200 permutations, we randomly shuffled the recorded EEG data across trials and repeated the MI calculation for each sensor and time point. We computed the maximum of the resulting 3D CMI matrix (time vs. EEG sensors vs. left and right eye visibility) for each permutation. For each observer we used the 99th percentile across permutations as the statistical threshold for each observer.

### Selection of LOT and ROT Sensors

As described above we calculated MI between EEG and visibility of each eye for each sensor and time point. We selected for further analyses the lateral occipito-temporal sensors with maximum MI for the eye contralateral to the sensor in a time window 100 and 200 ms post stimulus. On the left hemisphere, for LOT we selected the sensor with maximum right eye MI from sensors on the radial axes of P07, P7, and TP7 (excluding midline Oz and neighboring O1 radial axes). On the right hemisphere, for ROT we selected the sensor with maximum left eye MI from sensors on the radial axes of PO8, P8, TP8 (excluding midline Oz and neighboring O2 radial axes). This selection was necessary for simpler statistical analysis but, as indicated by the full topography plots in Figure 5*C* and Supplementary Figs. S2–S16, our results are robust to different methods of LOT and ROT sensor selection.

### Coding: MI Statistical Significance

We determined the statistical significance of left and right eye coding on LOT and ROT as follows. We compared the maximum CMI value within a window of 0 to 400 ms post-stimulus, to the 99th percentile over permutations (described above) of the maximum CMI over those time points, all electrodes, and both features. This resulted in a determination of whether there was any statistically significant coding during that time interval, corrected for multiple comparisons over all electrodes (necessary since we selected LOT and ROT based on MI values), time points and both left and right eye visibility.

### Temporal Precedence: Latency measures

Whereas a human observer can easily determine which of 2 time varying noisy signals leads the other, it is not straightforward to rigorously define and quantify this. Here, we are interested in the relative timings of the contralateral and ipsilateral eye CMI on LOT and ROT, rather than their amplitudes. We therefore first normalized the CMI curves to their maximum value (in the window 150–250 ms post-stimulus, as plotted in Fig. 4*A*,*B*). The variability of these curves makes any latency measure that depends on a specific time point problematic due to a lack of robustness with respect to the time point selected. To illustrate, consider the time courses in Figure 4*C* where a simple peak-to-peak measure would result in a value ~100 ms for the left eye (blue curves), and −30 ms for the right eye (red curves, grey dashed line shows ipsi-lateral peak before contra-lateral peak). These values do not sensibly reflect the actual relationship – for left eye the value seems too high, and for the right eye it is in the wrong direction; the solid contralateral curve appears to lead the dashed ipsilateral curve in the period 130–180 ms. Similar problems would affect any measure that compares 2 specific points.

Hence, we considered the latency of the CMI curves not over a single value, but over a range of *y*-axis values (Fig. 4*C*). We restricted our analysis to 100–300 ms post-stimulus, and considered normalized CMI values where both curves were significant. We split the range of normalized CMI values above the highest significance threshold into 100 values and calculated the mean latency between the 2 curves over these values (indicated by gray lines in Fig. 4*C*). This is equivalent to integrating the latency of the curves over the *y*-axis and normalizing by the *y*-axis range.

### Coding Equivalence: Redundancy

Redundancy (equivalent to negative interaction information Cover and Thomas, 1991) quantifies the CMI overlap between 2 variables—that is the amount of CMI about eye visibility that is common to both EEG responses. We calculated this as described in the main text and illustrated in Figure 5*B*:

$$\begin{array}{c} {\mathrm{Red}}_{S_{L}}\left(R_{\mathrm{LOT}};R_{\mathrm{ROT}}\right)=I\left(S_{L};R_{\mathrm{LOT}}|S_{R}\right)+I\left(S_{L};R_{\mathrm{ROT}}|S_{R}\right)\\ -I\left(S_{L};{R_{\mathrm{LOT}},R}_{\mathrm{ROT}}|S_{R}\right) \end{array}$$

$R_{\mathrm{LOT}}$/$R_{\mathrm{ROT}}$ represent the response across trials at each electrode at the appropriate CMI peak (here left eye). Redundancy is bounded above by each of the individual CMI values and the CMI between the two EEG responses (Cover and Thomas 1991). We therefore normalized by the minimum of these 3 quantities:

$$\frac{{\mathrm{Red}}_{S_{L}}\left(R_{\mathrm{LOT}};R_{\mathrm{ROT}}\right)}{\min[I\left(S_{L};R_{\mathrm{LOT}}|S_{R}\right),I\left(S_{L};R_{\mathrm{ROT}}|S_{R}\right),I\left(R_{\mathrm{ROT}};R_{\mathrm{LOT}})\right]}$$

We calculated redundancy between LOT and ROT for each eye feature at the time of the peak of the CMI timecourse (within 50–250 ms; Table 1).

**Table 1:** Time of MI peak used for redundancy calculation. Median (min max) in ms

	Left eye	Right eye
ROT	167 (140 188)	180 (156 246)
LOT	177 (148 216)	162 (140 180)

For the redundancy topography plots in Figure 5*C* and Supplementary Figs. S2–S16 we fixed the contralateral electrode and CMI peak time as above (i.e., ROT for left eye, LOT for right eye) as a seed response. For each eye, we then calculated the redundancy between this seed response and every other sensor and time point where there was significant CMI about that eye (*P* = 0.01 with multiple comparison correction as described above).

We also computed CMI directly between $R_{\mathrm{LOT}}$ and $R_{\mathrm{ROT}}$ conditioning out any variation due to either stimulus: $I\left(R_{\mathrm{ROT}};R_{\mathrm{LOT}}{|S}_{R}{,S}_{L}\right)$. Since it is not clear how best to define a permutation scheme in this case, we performed 1000 bootstrap samples (resampling with replacement), and took the 0.5th percentile as the lower bound of a 99% confidence interval.

## Supplementary Material

Supplementary material can be found at: http://www.cercor.oxfordjournals.org/.

## Funding

A BBSRC DTP (WestBio) Studentship (K.J.); the Wellcome Trust (098433, 107802) (J.G. and P.G.S.); the BBSRC (BB/J018929/1) (G.A.R., P.G.S., and NJvR); the Seventh Framework Programme for Research of the European Commission (FP7-ICT-2011.9.11; VISUALISE FP7-600954) (S.P.).

## Supplementary Material

Supplementary Data
