# Supplementary material for: The Deceptively Simple N170 Reflects Network Information Processing Mechanisms Involving Visual Feature Coding and Transfer Across Hemispheres
Source: Cereb Cortex. 2016 Oct 17;26(11):4123–35. doi: 10.1093/cercor/bhw196 (PMC5066825; doi:10.1093/cercor/bhw196)
Supplement: Supplementary Data [file supp_26_11_4123__index.html]

The Deceptively Simple N170 Reflects Network Information Processing Mechanisms Involving Visual Feature Coding and Transfer Across Hemispheres — The Deceptively Simple N170 Reflects Network Information Processing Mechanisms Involving Visual Feature Coding and Transfer Across Hemispheres — Supplementary Data 

# The Deceptively Simple N170 Reflects Network Information Processing Mechanisms Involving Visual Feature Coding and Transfer Across Hemispheres

## Supplementary Data

Supplementary Data

- Supplementary Data - tiff file
- Supplementary Data - tiff file
- Supplementary Data - tiff file
- Supplementary Data - tiff file
- Supplementary Data - tiff file
- Supplementary Data - tiff file
- Supplementary Data - tiff file
- Supplementary Data - tiff file
- Supplementary Data - tiff file
- Supplementary Data - tiff file
- Supplementary Data - tiff file
- Supplementary Data - tiff file
- Supplementary Data - tiff file
- Supplementary Data - tiff file
- Supplementary Data - tiff file
- Supplementary Data - tiff file
- Supplementary Data - docx file
