# Supplementary figures and images for: The Deceptively Simple N170 Reflects Network Information Processing Mechanisms Involving Visual Feature Coding and Transfer Across Hemispheres
Source: Cereb Cortex. 2016 Oct 17;26(11):4123–35. doi: 10.1093/cercor/bhw196 (PMC5066825; doi:10.1093/cercor/bhw196)

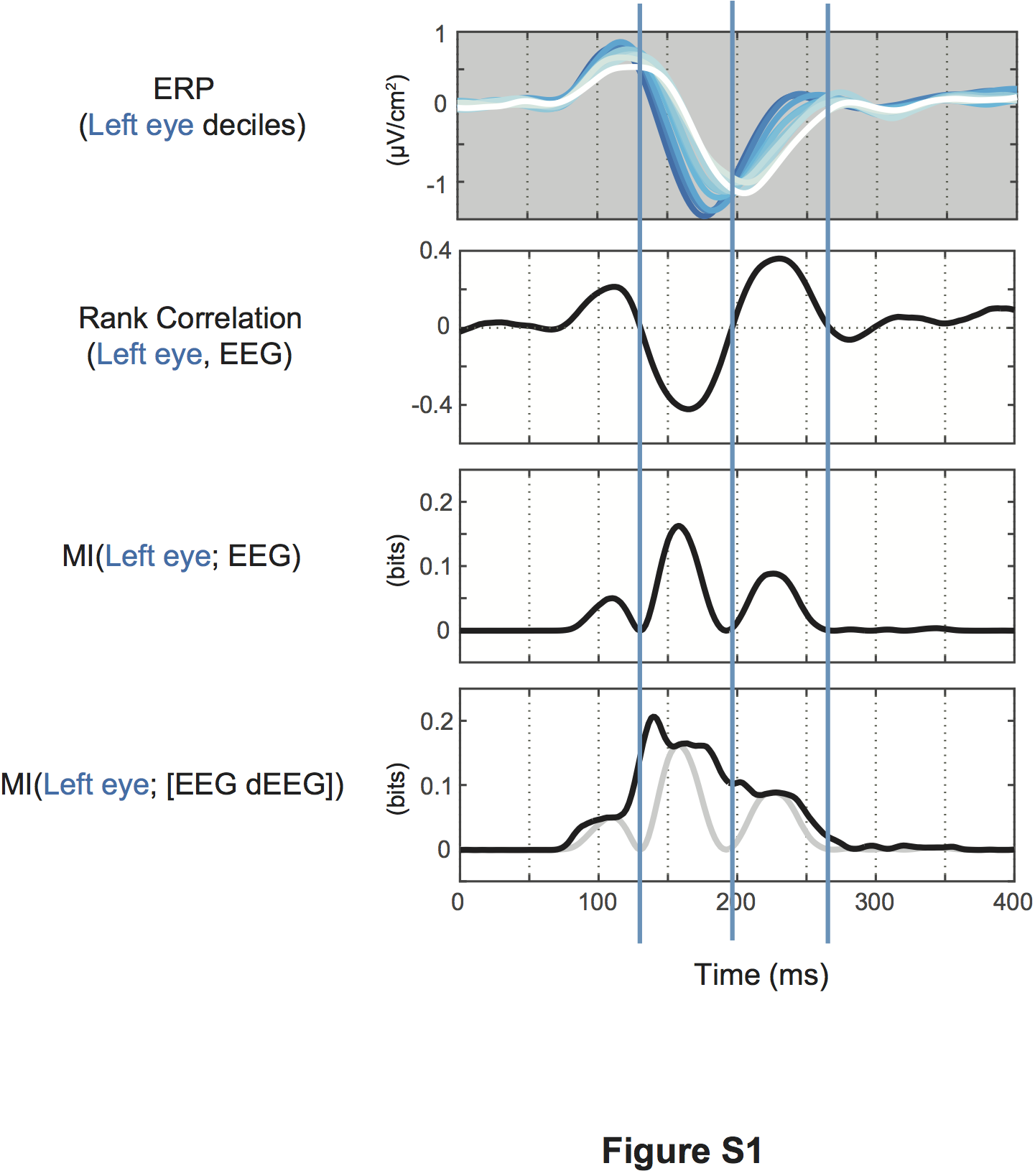

Supplement: Supplementary Data [file supp_bhw196_S1_Figure.tif]

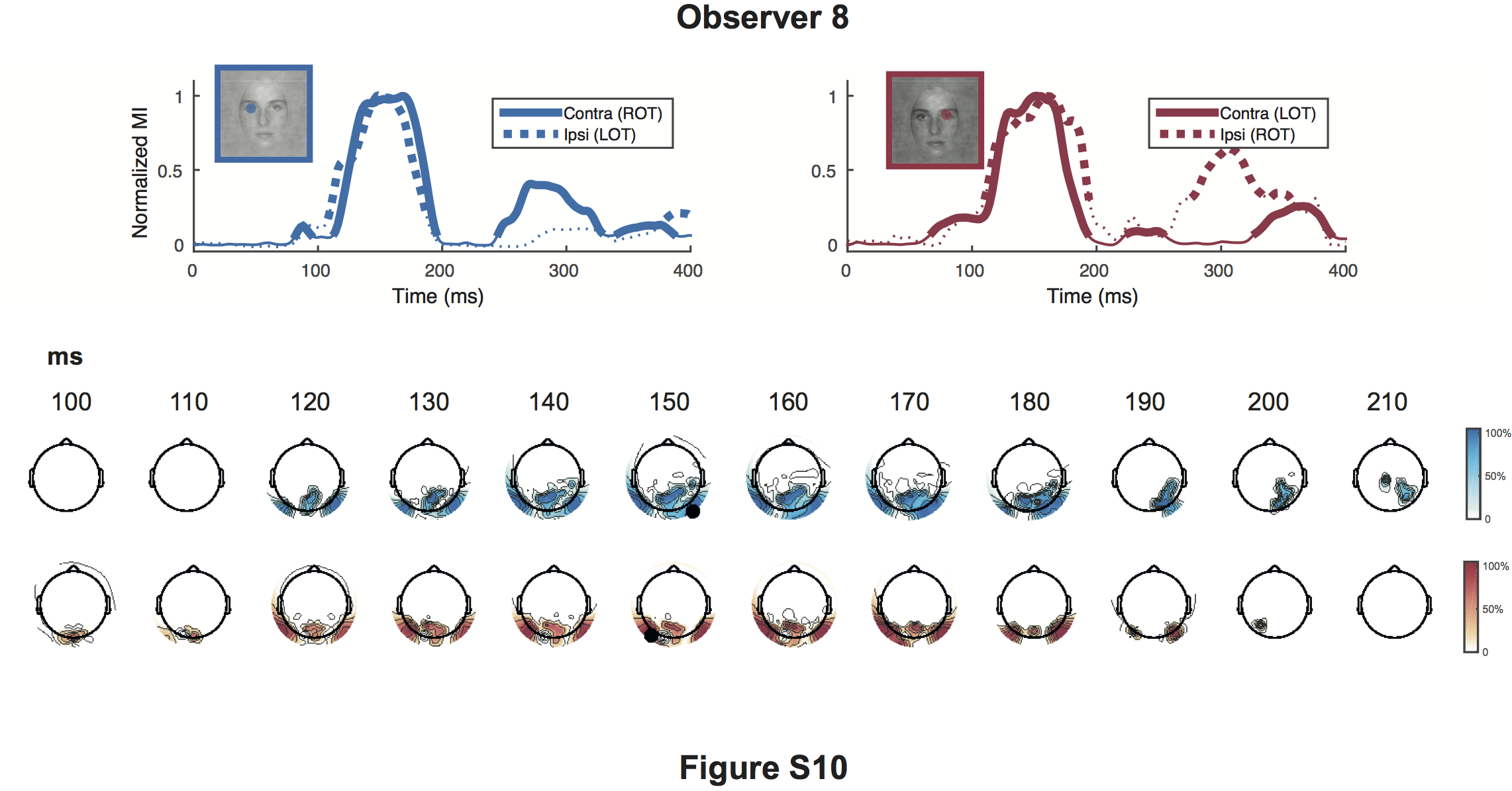

Supplement: Supplementary Data [file supp_bhw196_S10_Figure.tif]

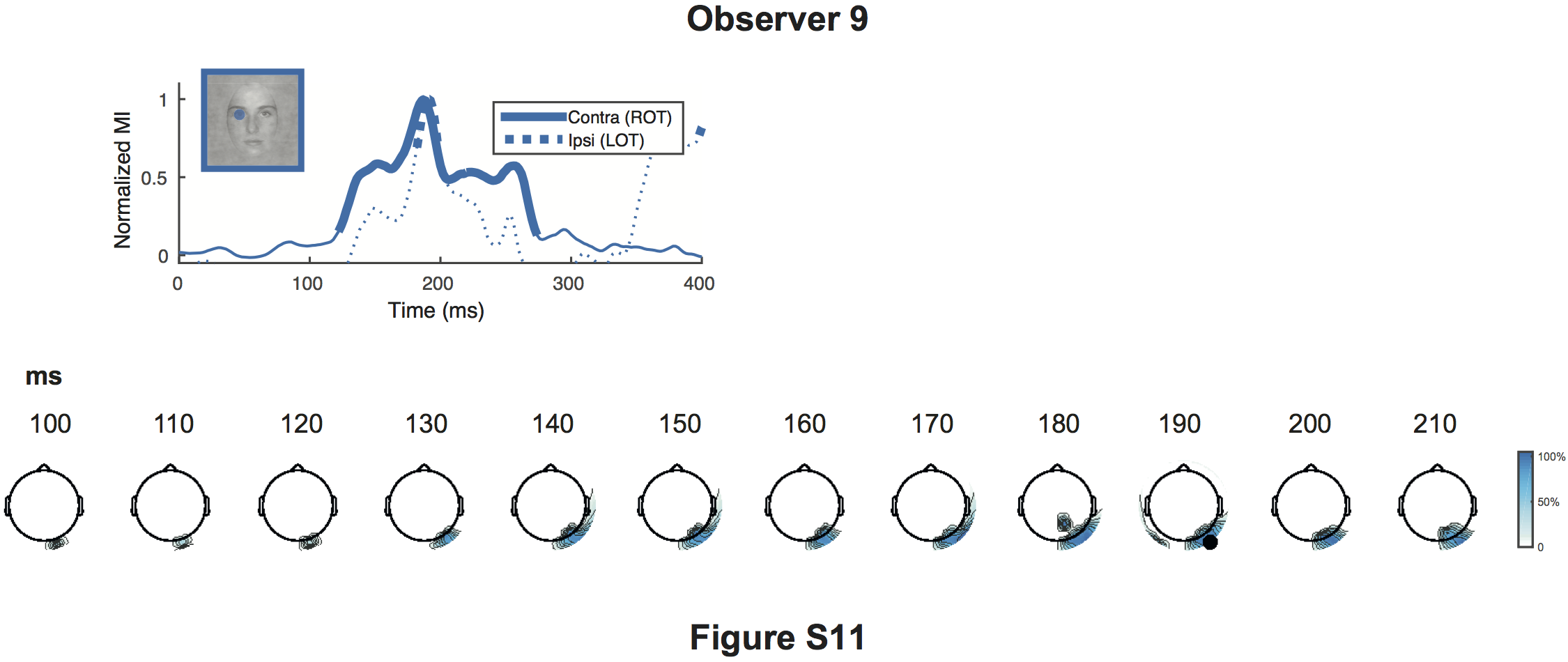

Supplement: Supplementary Data [file supp_bhw196_S11_Figure.tif]

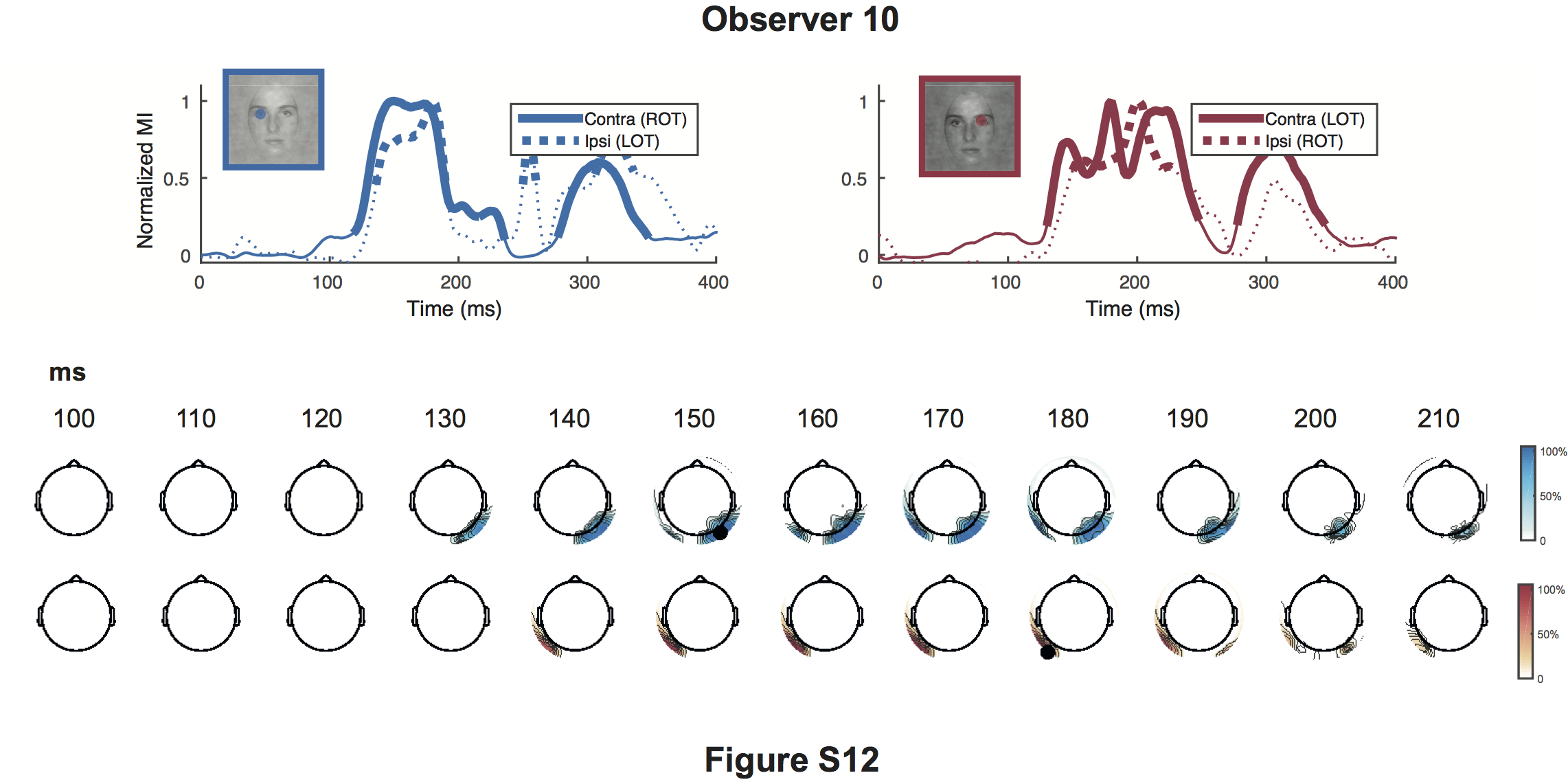

Supplement: Supplementary Data [file supp_bhw196_S12_Figure.tif]

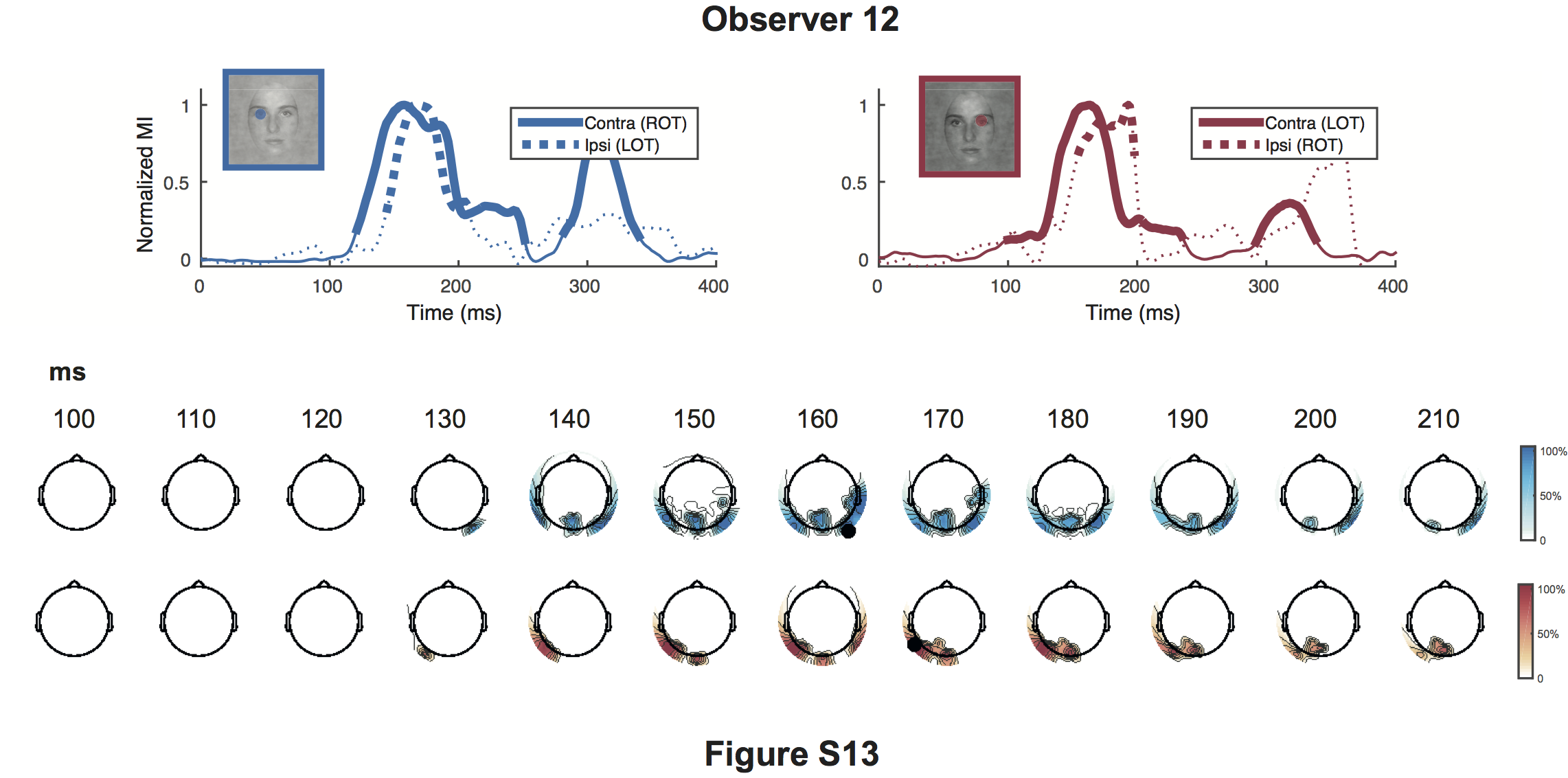

Supplement: Supplementary Data [file supp_bhw196_S13_Figure.tif]

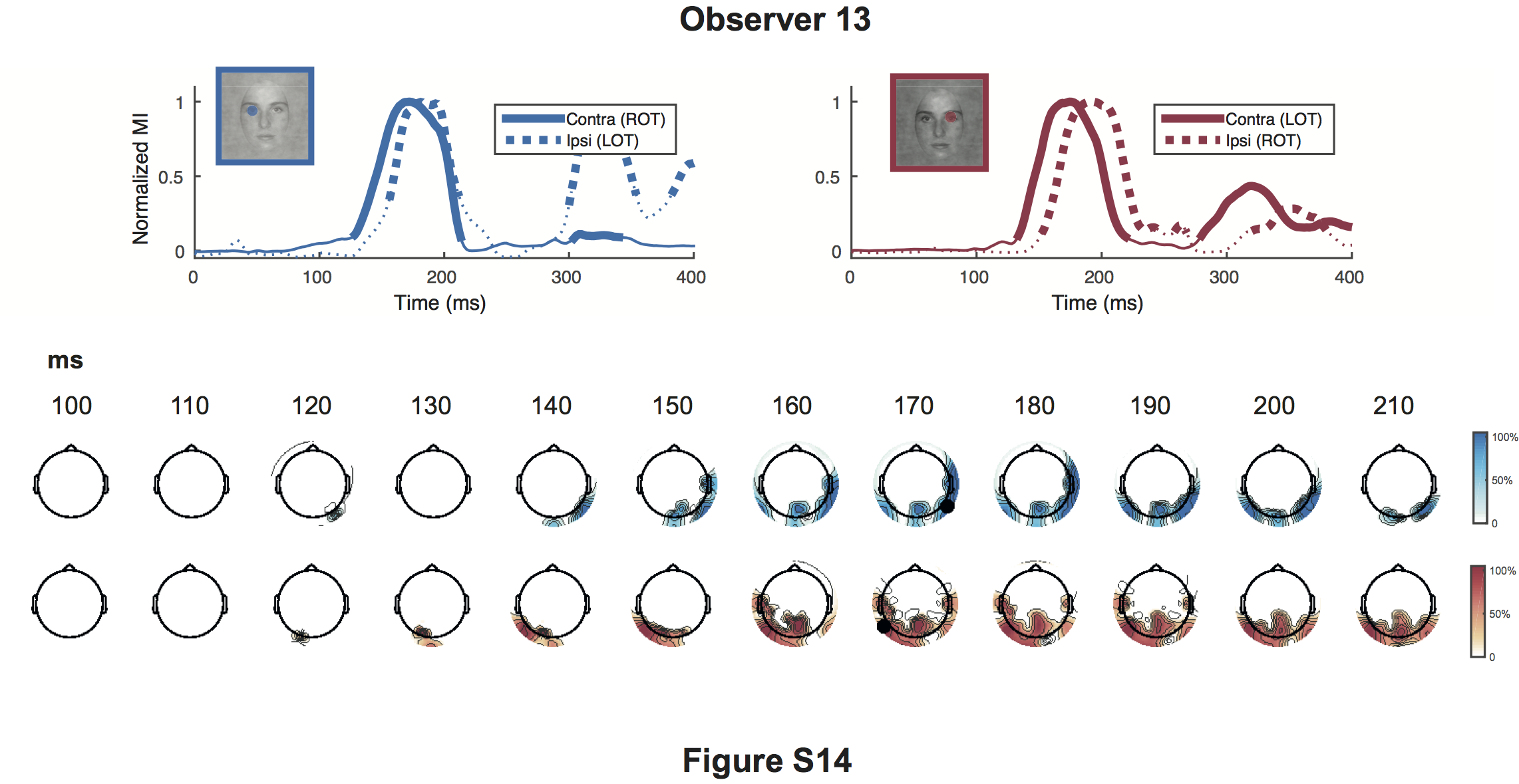

Supplement: Supplementary Data [file supp_bhw196_S14_Figure.tif]

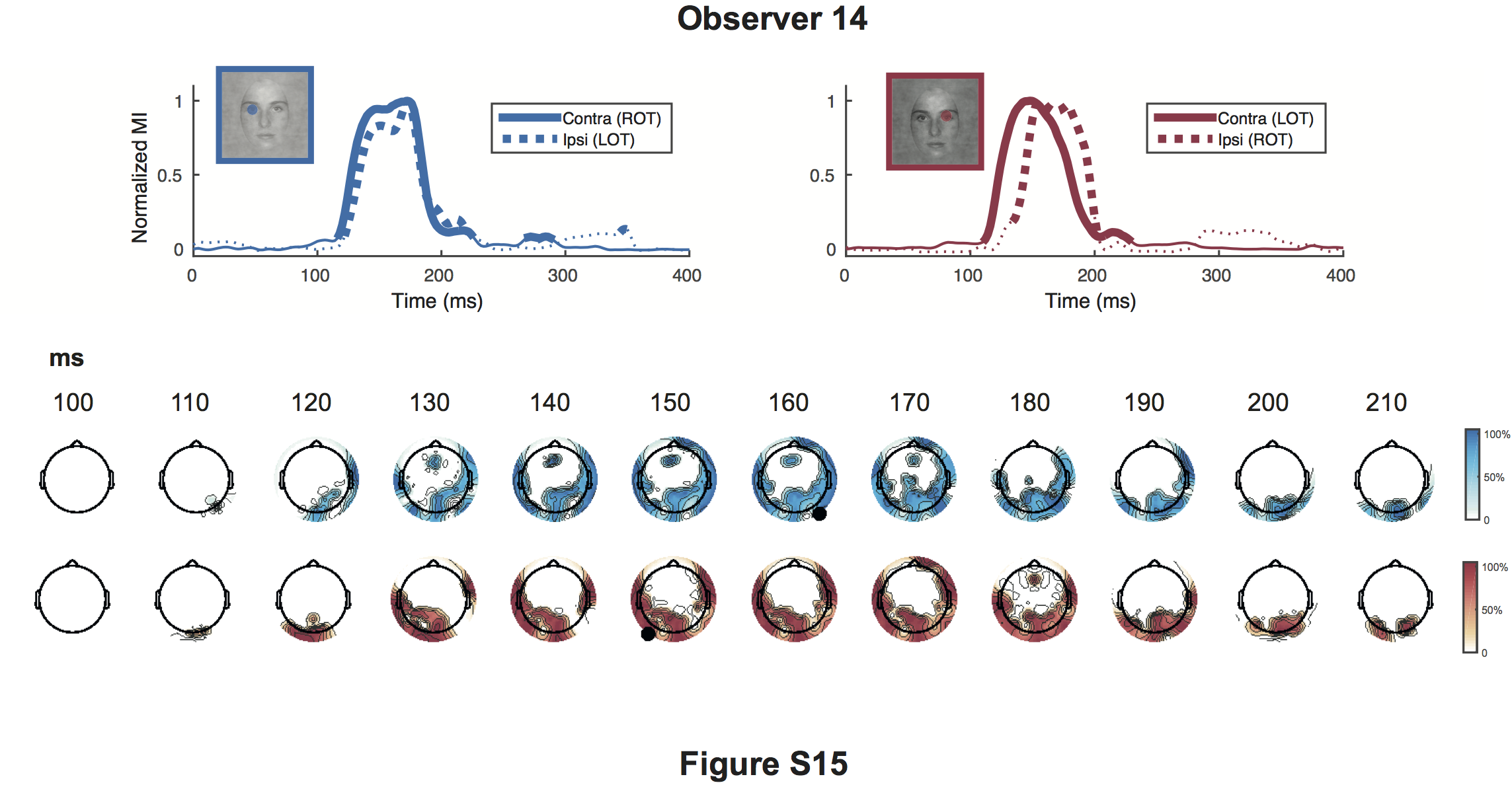

Supplement: Supplementary Data [file supp_bhw196_S15_Figure.tif]

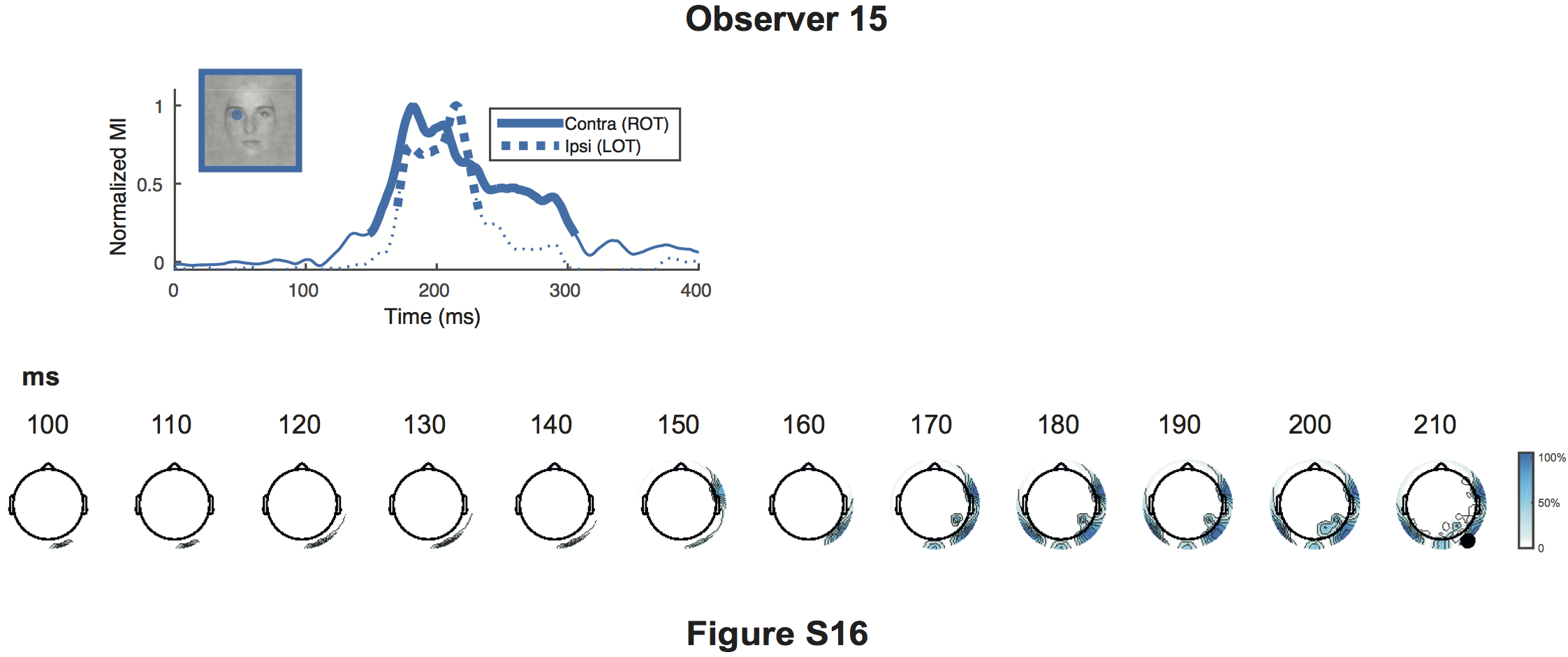

Supplement: Supplementary Data [file supp_bhw196_S16_Figure.tif]

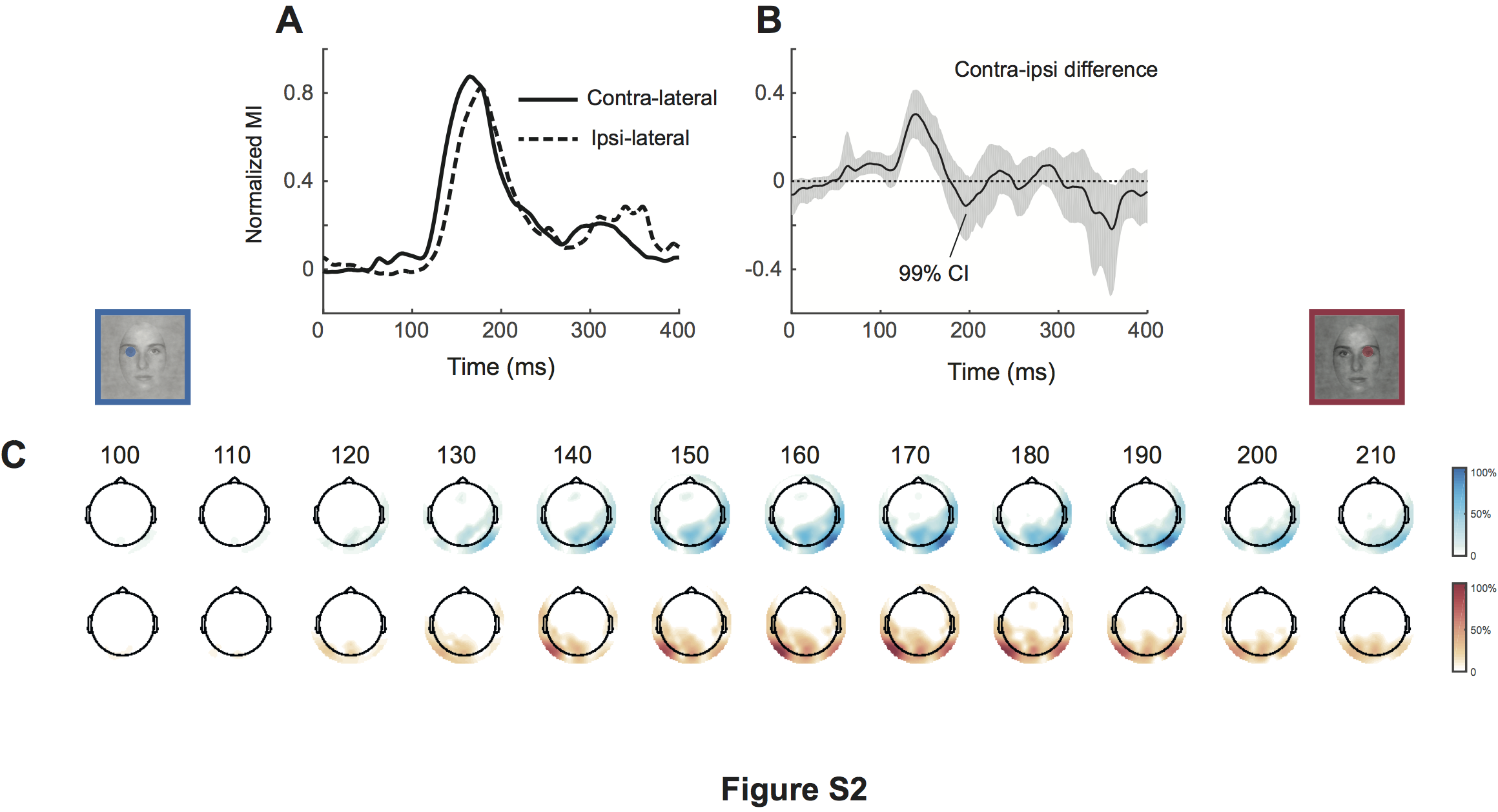

Supplement: Supplementary Data [file supp_bhw196_S2_Figure.tif]

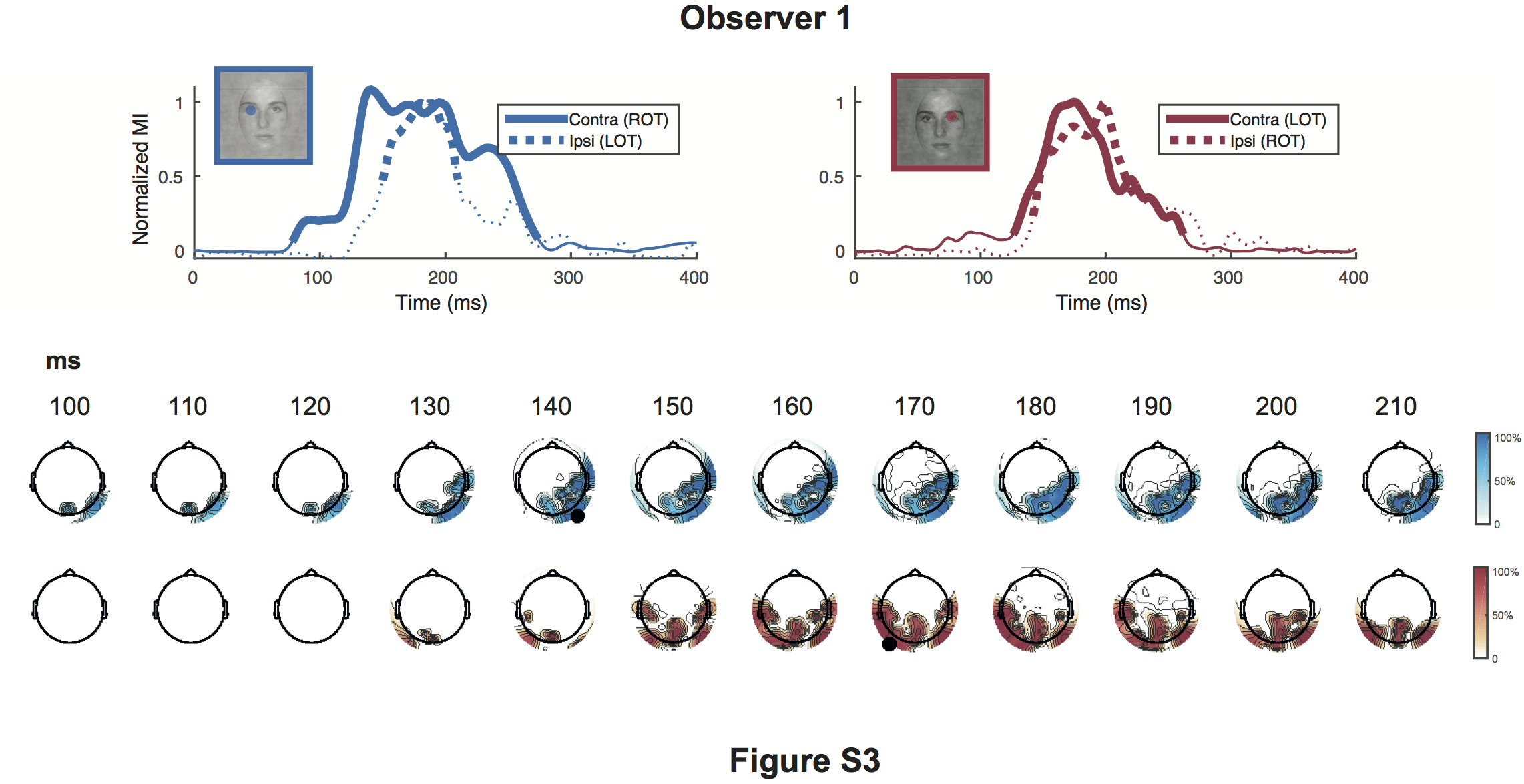

Supplement: Supplementary Data [file supp_bhw196_S3_Figure.tif]

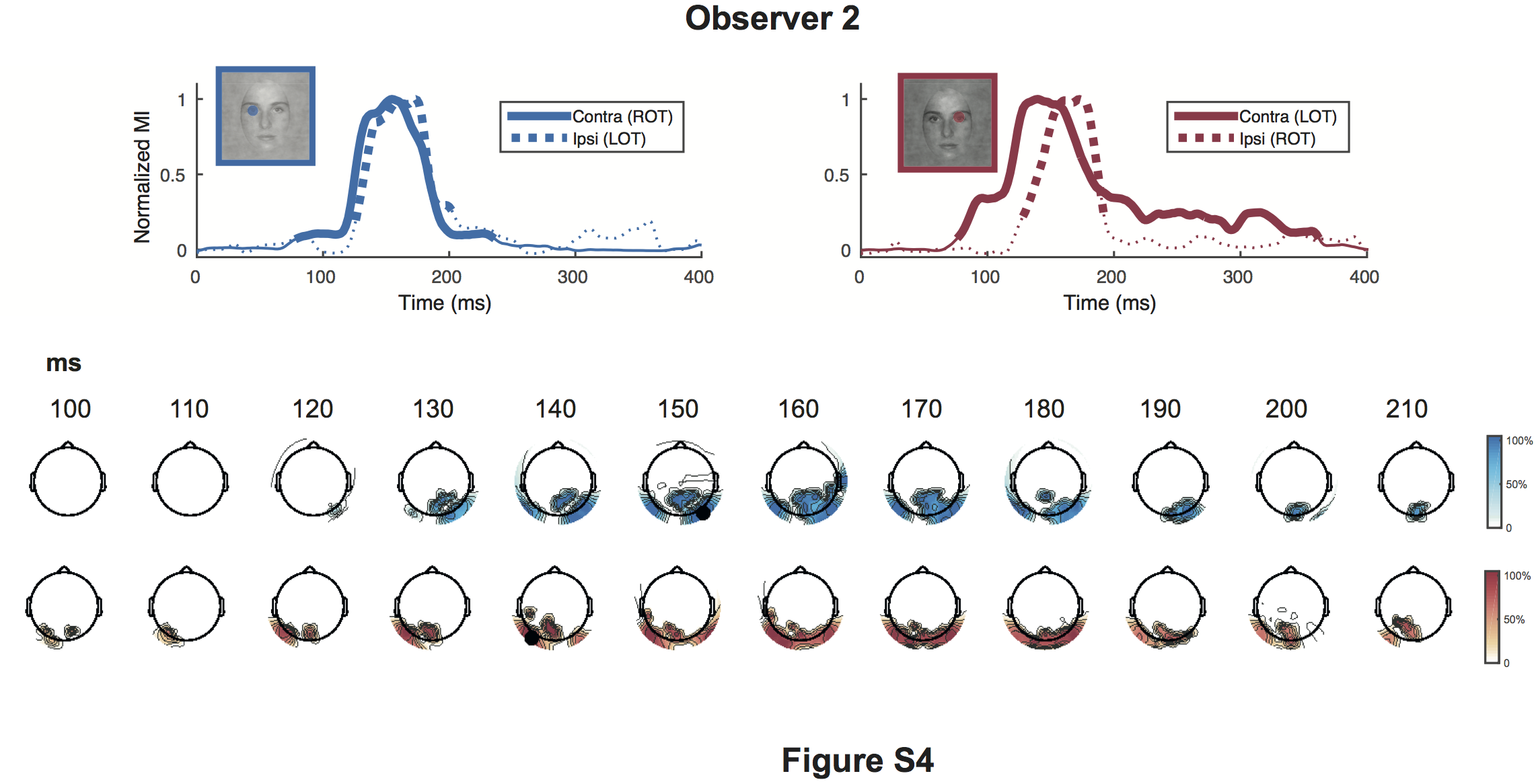

Supplement: Supplementary Data [file supp_bhw196_S4_Figure.tif]

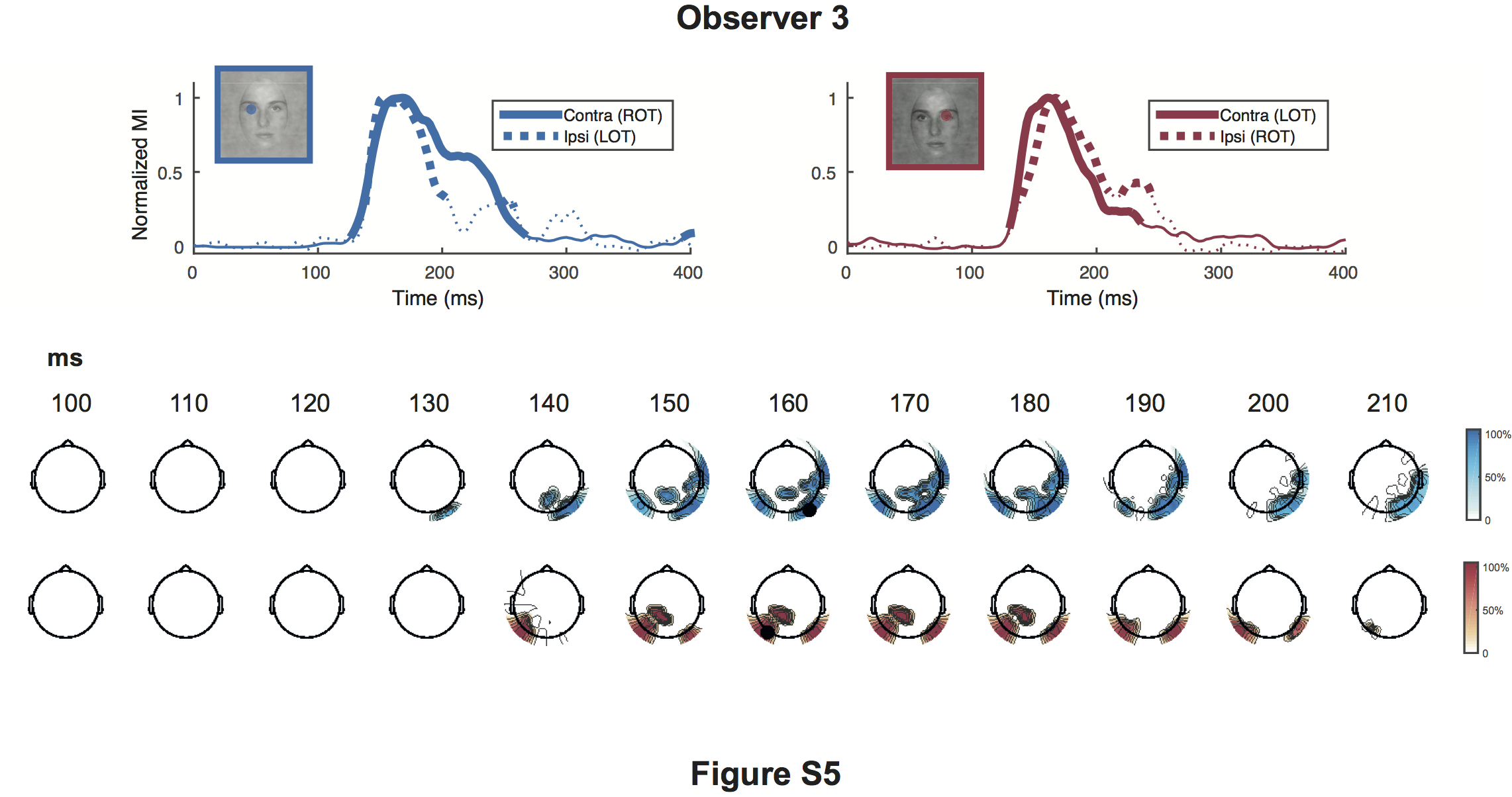

Supplement: Supplementary Data [file supp_bhw196_S5_Figure.tif]

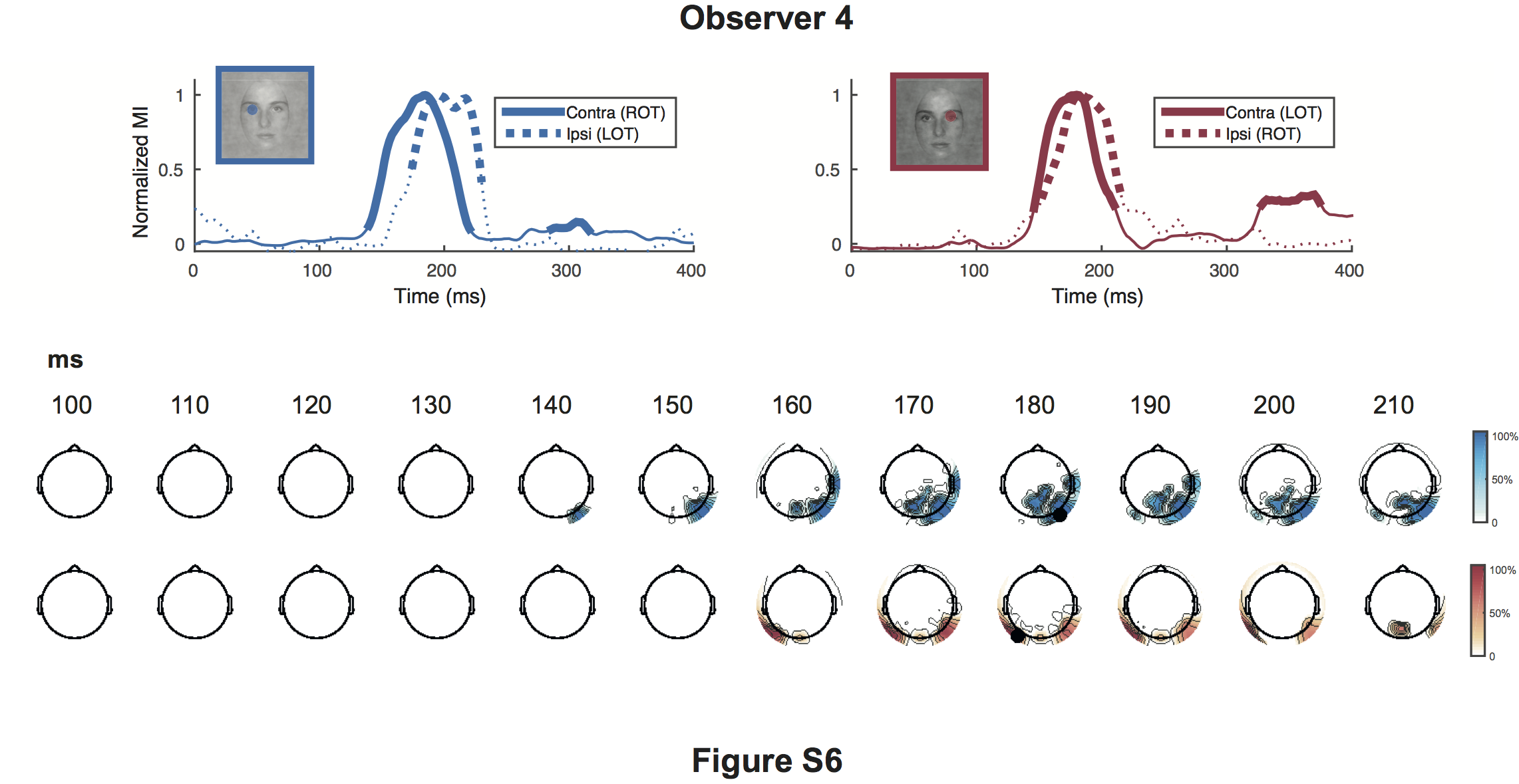

Supplement: Supplementary Data [file supp_bhw196_S6_Figure.tif]

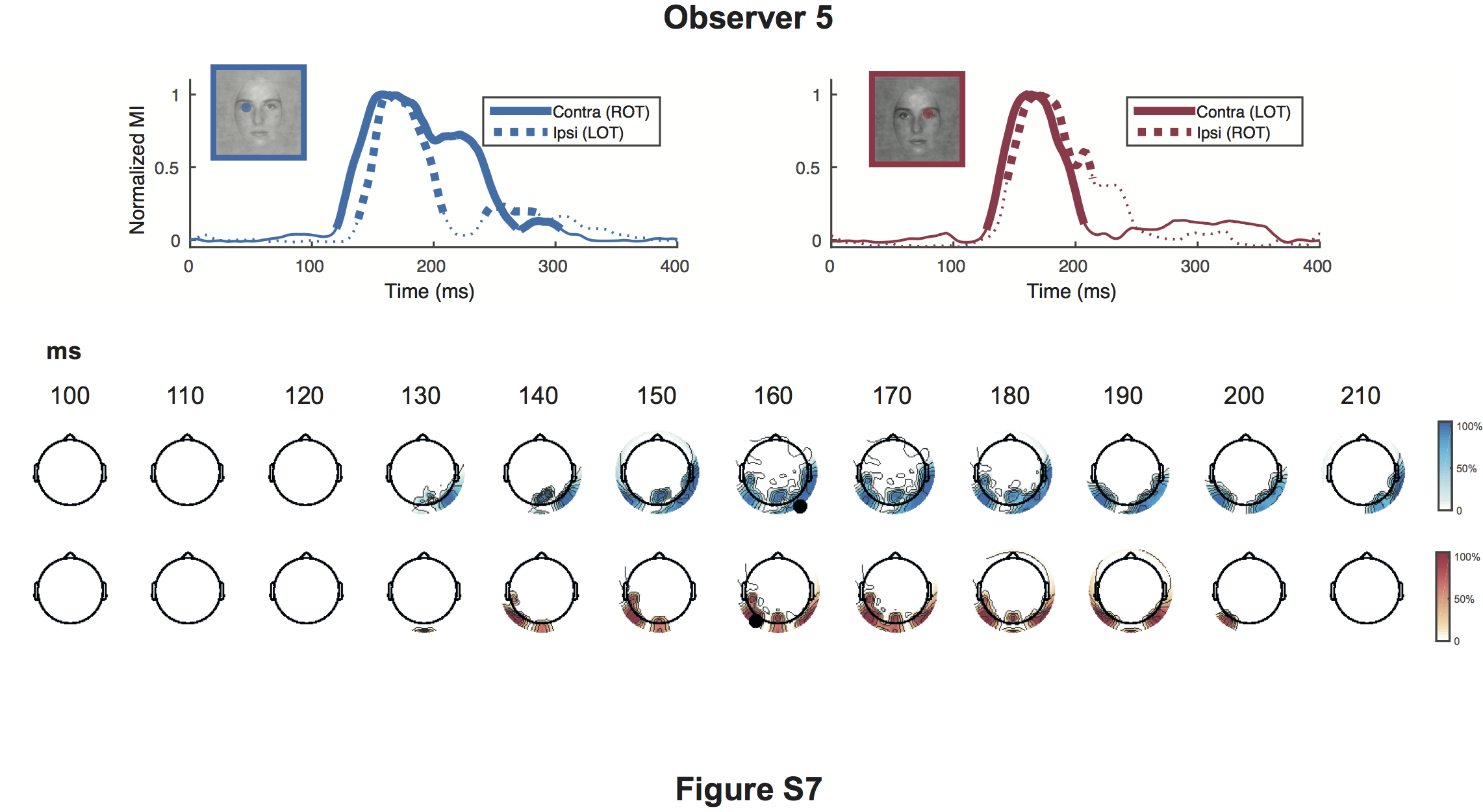

Supplement: Supplementary Data [file supp_bhw196_S7_Figure.tif]

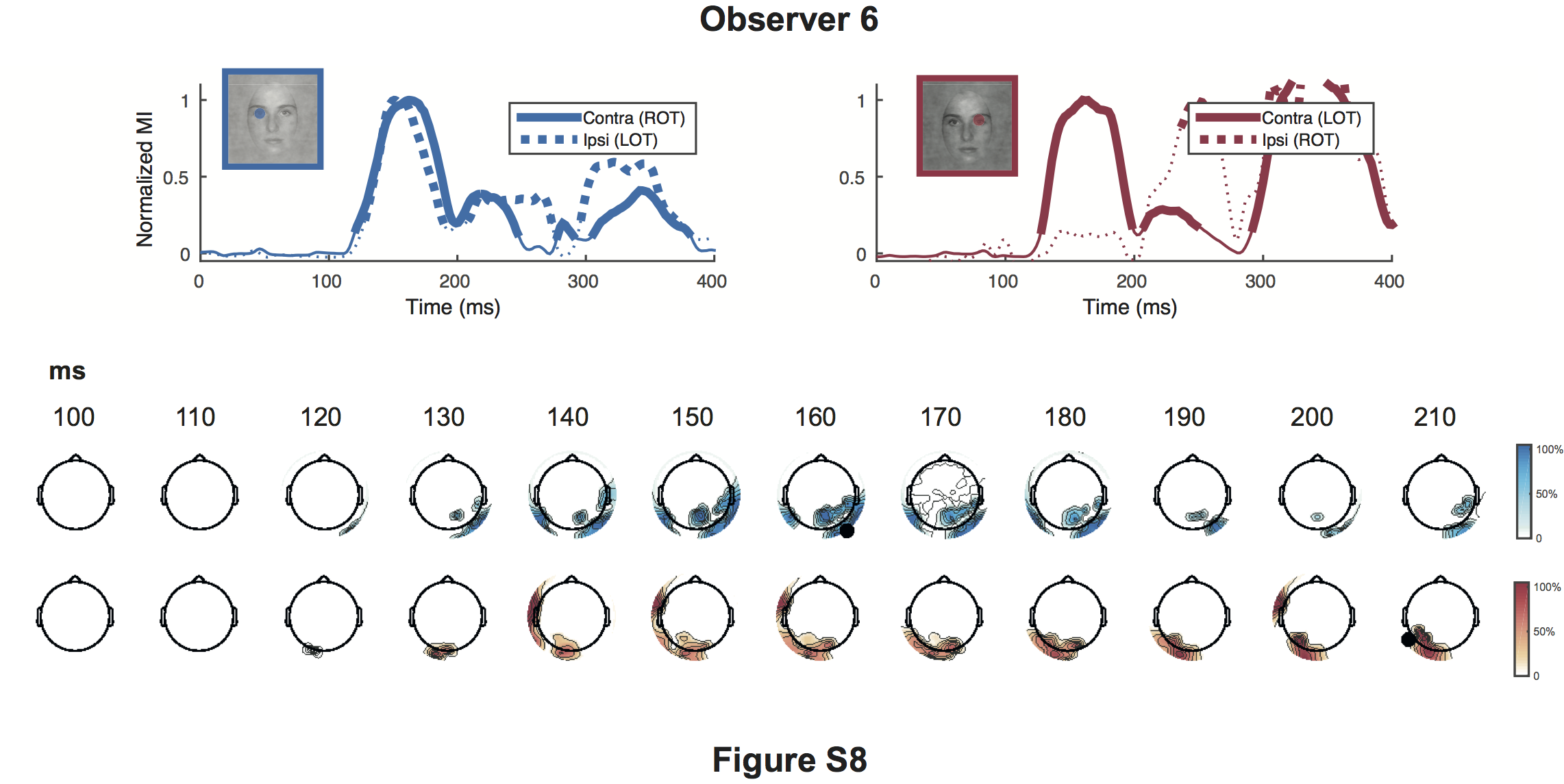

Supplement: Supplementary Data [file supp_bhw196_S8_Figure.tif]

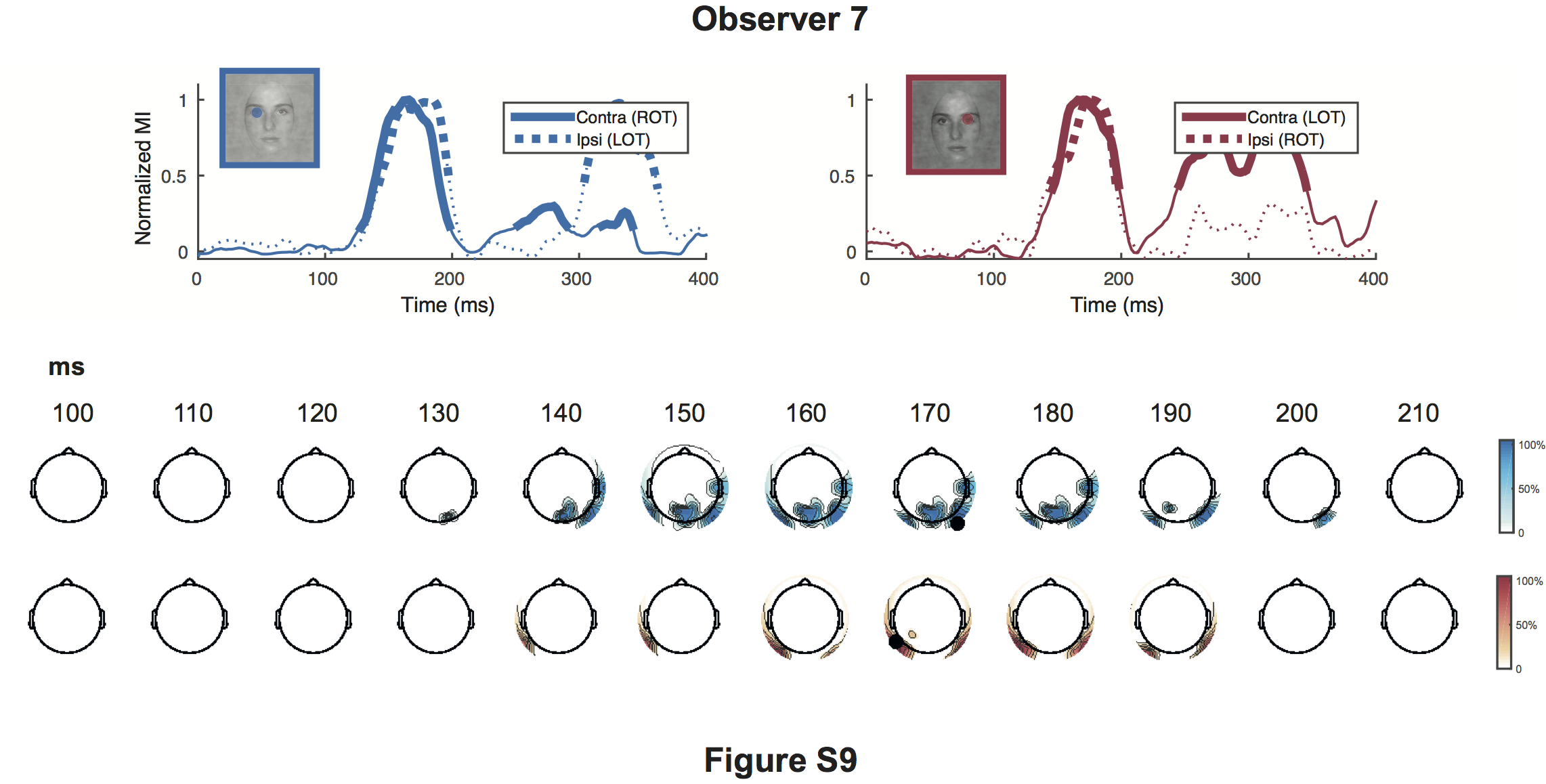

Supplement: Supplementary Data [file supp_bhw196_S9_Figure.tif]
