# Supplementary material for: The Deceptively Simple N170 Reflects Network Information Processing Mechanisms Involving Visual Feature Coding and Transfer Across Hemispheres
Source: Cereb Cortex. 2016 Oct 17;26(11):4123–35. doi: 10.1093/cercor/bhw196 (PMC5066825; doi:10.1093/cercor/bhw196)
Supplement: Supplementary Data [file supp_bhw196_Supplemental_Figure_Captions.docx]

# Supplemental Figure Captions

**Figure S1.** *Mutual information calculated on bivariate EEG response including temporal gradient.* From top to bottom, plots show (i) the ERP calculated from trials in each decile of left eye visibility (c.f. Figure 3) on ROT of observer 1, (ii) Spearman’s rank correlation between the left eye visibility and EEG voltage, calculated separately for each time point, (iii) MI between the left eye visibility and EEG voltage calculated separately for each time point and (iv) MI between left eye visibility and the 2D EEG response consisting of voltage and temporal gradient, calculated separately for each time point. Zero crossings of rank correlation, where the directionality of the voltage modulation changes, are indicated with vertical bars.

**Figure S2.** *Group average results for temporal precedence and coding equivalence.* **A.** Mean normalized MI time course over all instances with bilateral coding significance (observer and left or right eye, N=26 / 30) for contra- and ipsi-lateral sensors (LOT/ROT). **B**. Mean contra-ipsi difference over instances, with 99% bootstrap confidence interval. **C.** Mean normalized redundancy topography sequences calculated as described in the text.

**Figures S3-S16.** *Individual observer results for temporal precedence and coding equivalence.* Time courses show the normalized MI time courses for the left eye visibility (blue, left plot) and right eye visibility (red, right plot) on the contra-lateral (solid line) and ipsi-lateral sensors (dashed line). Topography sequences show normalized redundancy (seed sensor and time indicated with black circle).
